# Friend or foe: divergent immunomodulatory effects of metabolites derived from the virucidal zinc finger inhibitor SAMT-247

**DOI:** 10.3389/fimmu.2026.1879043

**Published:** 2026-08-20

**Authors:** Emmanuel K. Woode, Katherine C. Goldfarbmuren, Oluwatobi T. Arisa, Massimiliano Bissa, Luca Schifanella, Marco Robello, Manjula Gunawardana, John A. Moss, Amalia E. Castonguay, Irina Butkyavichene, Lisa M. Jenkins, Earl B. Ettienne, Daniel H. Appella, Marc M. Baum, William D. Figg, Genoveffa Franchini, Mohammad Arif Rahman

**Affiliations:** 1 Animal Models and Retroviral Vaccines Section, Basic Research Laboratory, Center for Cancer Research, National Cancer Institute, National Institutes of Health, Bethesda, MD, United States; 2 Advanced Biomedical Computational Science, Frederick National Laboratory for Cancer Research, Leidos Biomedical Research, Inc., Frederick, MD, United States; 3 Center for Cancer Research Collaborative Bioinformatics Resource, National Cancer Institute, National Institutes of Health, Bethesda, MD, United States; 4 Genitourinary Malignancies Branch, Center for Cancer Research, National Cancer Institute, National Institutes of Health, Bethesda, MD, United States; 5 Synthetic Bioactive Molecules Section, National Institute of Diabetes and Digestive and Kidney Diseases, National Institutes of Health, Bethesda, MD, United States; 6 Department of Chemistry, Oak Crest Institute of Science, Monrovia, CA, United States; 7 Mass Spectrometry Resource, Laboratory of Cell Biology, National Cancer Institute, National Institutes of Health, Bethesda, MD, United States; 8 College of Pharmacy, Howard University College of Pharmacy, Washington, DC, United States

**Keywords:** HIV/SIV, HIV/SIV vaccine, immunomodulation, metabolite of SAMT-247, mucosal immunity, SAMT-247, vaccine-induced immune responses, zinc finger inhibitor

## Abstract

**Background:**

Topical administration of SAMT-247, a mercaptobenzamide thioester zinc finger inhibitor targeting the HIV nucleocapsid Gag zinc finger protein, generates multiple metabolites in mucosal tissues, including Met-A, Met-B, Met-C, and Met-D. Prior studies established that SAMT-247, delivered either as a vaginal gel or via an intravaginal ring (IVR), synergizes with the ΔV1DNA/ALVAC-SIV/ΔV1gp120/alum vaccine regimen to markedly reduce vaginal SIV_mac251_ acquisition risk. This enhanced protection is associated with augmented protective mucosal immunity and reduced inflammatory responses that promote viral acquisition.

**Methods:**

We performed *in vivo* characterization of SAMT-247 metabolite distribution in the vaginal compartment of macaques and evaluate the biological activity of individual metabolites using *ex vivo* rectal mucosal biopsies from vaccinated animals.

**Results:**

High concentrations of Met-B, Met-C, and Met-D were detected in vaginal secretions, whereas vaginal tissues contained predominantly Met-D, low levels of Met-A, and no detectable Met-B or Met-C. Functionally, Met-D most closely recapitulated and reinforced the protective immune profile associated with SAMT-247, preserving or expanding IL-17^+^NKp44^+^ innate lymphoid cells (ILCs) and CD107a^+^NKG2A^+^ natural killer (NK) cells, increasing CD73^+^ ILCs, NK and dendritic cell as well as IL-10^+^ dendritic cell and monocyte populations, and reducing inflammatory TNF-α-producing myeloid subsets. Met-B and Met-C partially reproduced this profile, enhancing CD73^+^ NK/ILC populations and promoting anti-inflammatory myeloid responses, but with more limited effects on antiviral NK/ILC activity and attenuated NKp44^+^ ILC responses relative to Met-D. In contrast, Met-A, which lacks virucidal activity, failed to induce regulatory CD73^+^ and IL-10^+^ responses and diminished both protective IL-17^+^NKp44^+^ ILCs and cytolytic CD107a^+^NKG2A^+^ NK cells, consistent with inflammatory skewing. In vaccinated macaques receiving SAMT-247-releasing IVRs, plasma Met-A showed limited correlation with rectal mucosal immune responses, partially supporting the *ex vivo* findings.

**Conclusion:**

We observed distinct immunomodulatory effects associated with different SAMT-247 metabolites. These findings may guide future delivery strategies that favor tissue-available Met-D while limiting Met-A accumulation. More broadly, this study underscores the importance of metabolite-specific analyses for defining the biological activity and mechanisms of action of therapeutic agents.

## Introduction

Although antiretroviral therapy (ART) has transformed HIV treatment globally, only 77% of all people living with HIV (31.6 million people) have access to ART (1). Women account for more than half of all new HIV infections worldwide (1), and heterosexual transmission occurs predominantly across the female genital mucosa, where the local immune environment plays a critical role in determining susceptibility (2). Women and girls continue to bear a substantial share of the global HIV burden, accounting for 44% of all new HIV acquisitions and 53% of all people living with HIV in 2025 (3). Despite significant advances in HIV vaccine development (4–7), the generation of a protective and durable vaccine remains a major challenge (8–10). Pre-exposure prophylaxis (PrEP) has also emerged as an important prevention tool (11, 12), yet additional strategies are urgently needed to address limited PrEP accessibility, particularly among women and adolescent girls.

Topical anti-HIV microbicides with direct-acting antiretroviral properties, can be formulated as gels, ointments, creams, inserts (including suppositories, tablets, or ovules), films, or intravaginal rings. These products are applied to the vagina or rectum with the aim of preventing the sexual transmission of HIV in women and men (13–16). To date, the dapivirine intravaginal ring (DPV-VR) remains the only approved vaginally delivered microbicide with limited availability in 12 African countries (17), reflecting the limited success of other candidates in demonstrating adequate efficacy (18, 19). One promising microbicide candidate in the pre-clinical setting is the zinc finger protein inhibitor SAMT-247 (2-mercaptobenzamide thioester) (20), which has demonstrated efficacy *in vitro* against multiple HIV strains, including multidrug-resistant isolates (21). *In vivo*, SAMT-247 prevented vaginal SHIV (Simian-human immunodeficiency virus) infection in five of six female rhesus macaques challenged with a mixed R5/X4 virus (22). However, SAMT-247 failed to confer protection in two independent cohorts of animals when administered as gel 4 hours prior to SIV_mac251_ challenge (7, 23). Notably, vaginal administration of SAMT-247 in HEC gel substantially improved the effectiveness of the intramuscular ΔV1DNA/ALVAC-SIV/ΔV1gp120/alum vaccine regimen, boosting protection from 65% to 93.4% when applied prior to each SIV_mac251_ exposure (7). In a subsequent study using an intravaginal ring that continuously released a lower dose of SAMT-247, the compound alone showed a trend toward protection compared with naïve controls, and when combined with the ΔV1DNA/ALVAC-SIV/ΔV1gp120/alum regimen, resulted in decreased risk of viral acquisition by 82.8% (24).

**Figure 1:**
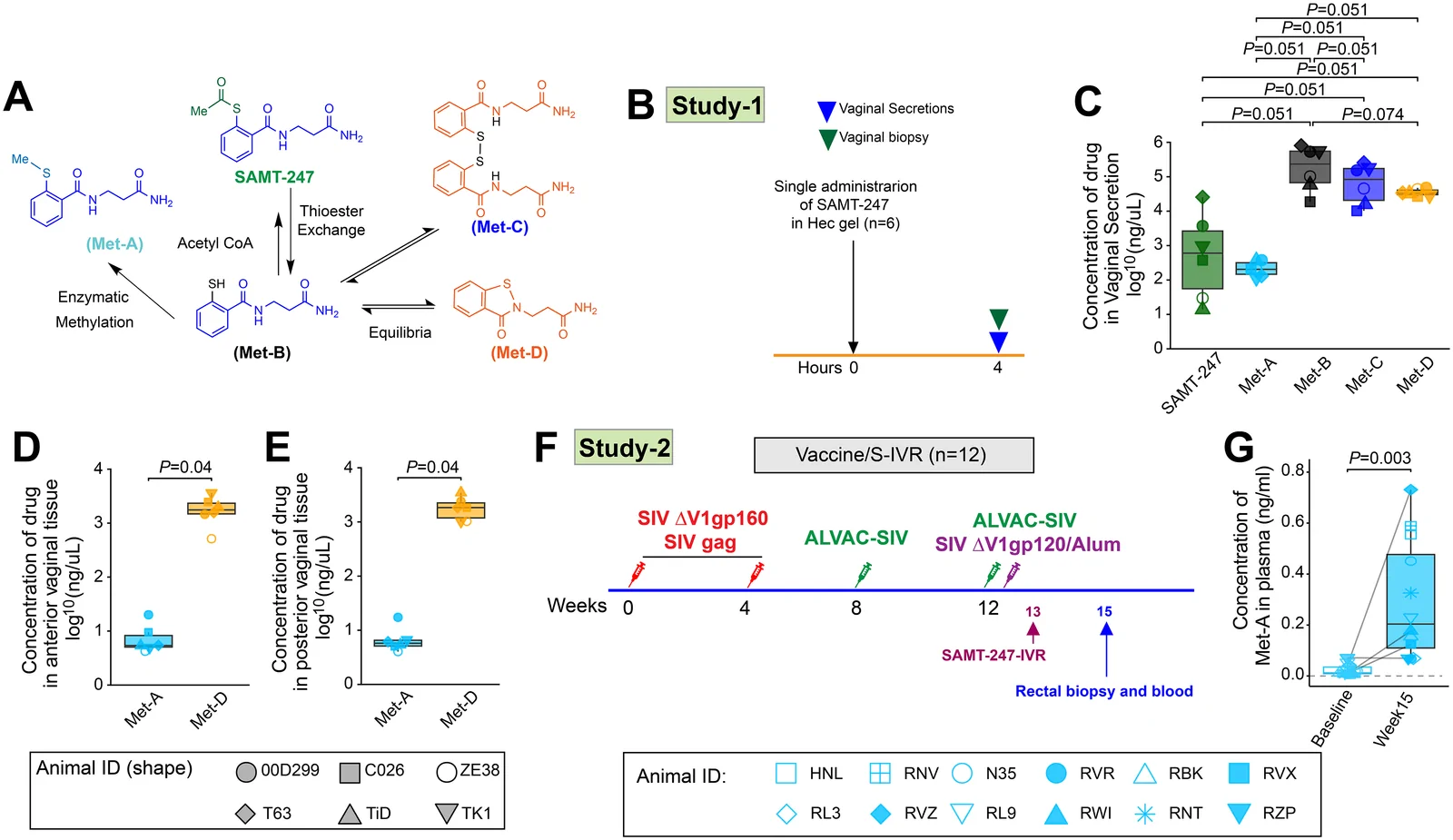
SAMT-247 metabolites and their distribution in the mucosa. **(A)** Metabolic pathway of SAMT-247 and formation of its derivatives. **(B)** Six macaques received a single intravaginal dose of 2 mL SAMT-247 gel (0.8% SAMT-247 in HEC). Four hours later, vaginal swabs and biopsies were collected for metabolite analysis. **(C)** Concentrations of SAMT-247 and metabolites detected in vaginal secretions. **(D, E)** Concentration of Met-A and Met-D in anterior and posterior vaginal tissue, respectively. **(F)** Twelve female macaques were primed with DNA-SIV and boosted with ALVAC-SIV, with or without ΔV1gp120 protein formulated in alum at designated time points. At week 13, animals received an intravaginal ring (IVR) designed for sustained release of SAMT-247. Rectal mucosal tissues and plasma samples were collected at week 15 for analysis. **(G)** Comparison of concentration of Met-A in plasma before and after insertion of IVR releasing SAMT-247 in the vagina. Data were analyzed using two-sided paired Wilcoxon tests. Box-and-whisker plots show minimum, Q1, median, Q3, and maximum values.

Further analyses indicated that SAMT-247 not only facilitates the generation of inactivated virions (20) but also modulates host immune responses (7, 24). Despite these encouraging findings, the immunomodulatory roles of its metabolites remain undefined. Here, we examine SAMT-247 and its metabolites to assess their contributions to immune modulation. SAMT-247 undergoes metabolic conversion after topical mucosal application, generating metabolites (Met-A, Met-B, Met-C, and Met-D, **Figure 1A**) that may have distinct immunological effects. Among these metabolites, the S-methyl derivative, Met-A, has been described as inactive against HIV (20, 25, 26), whereas SAMT-247, Met-C and Met-D, play a role to generate inactive HIV virions (20). Defining the role of these metabolites influencing mucosal innate responses, particularly those of protective NK cells/Innate lymphoid cells (ILCs) (7, 24, 27–29), DCs (6, 24, 27), and monocytes (7, 24), is critical for understanding the protective environment produced by the combined vaccine+SAMT-247 approach.

In this study, we found that SAMT-247 metabolites exhibit distinct immunomodulatory profiles. Met-A broadly reduced protective NK/ILC, dendritic cell, and monocyte responses and promoted a pro-inflammatory phenotype. In contrast, Met-D preserved or enhanced SAMT-247-associated immunomodulatory activity across NK/ILCs, mDCs, CD73^+^ mDCs, DC10, IL-10^+^ DCs, and IL-10^+^ monocytes, fostering an anti-inflammatory mucosal environment. These data suggest that minimizing the formation of the Met-A metabolite could further improve the ability of SAMT-247 to modulate immunity that protect against HIV acquisition.

## Results

### Study design and experimental framework for SAMT-247 assessment

**Figure 2:**
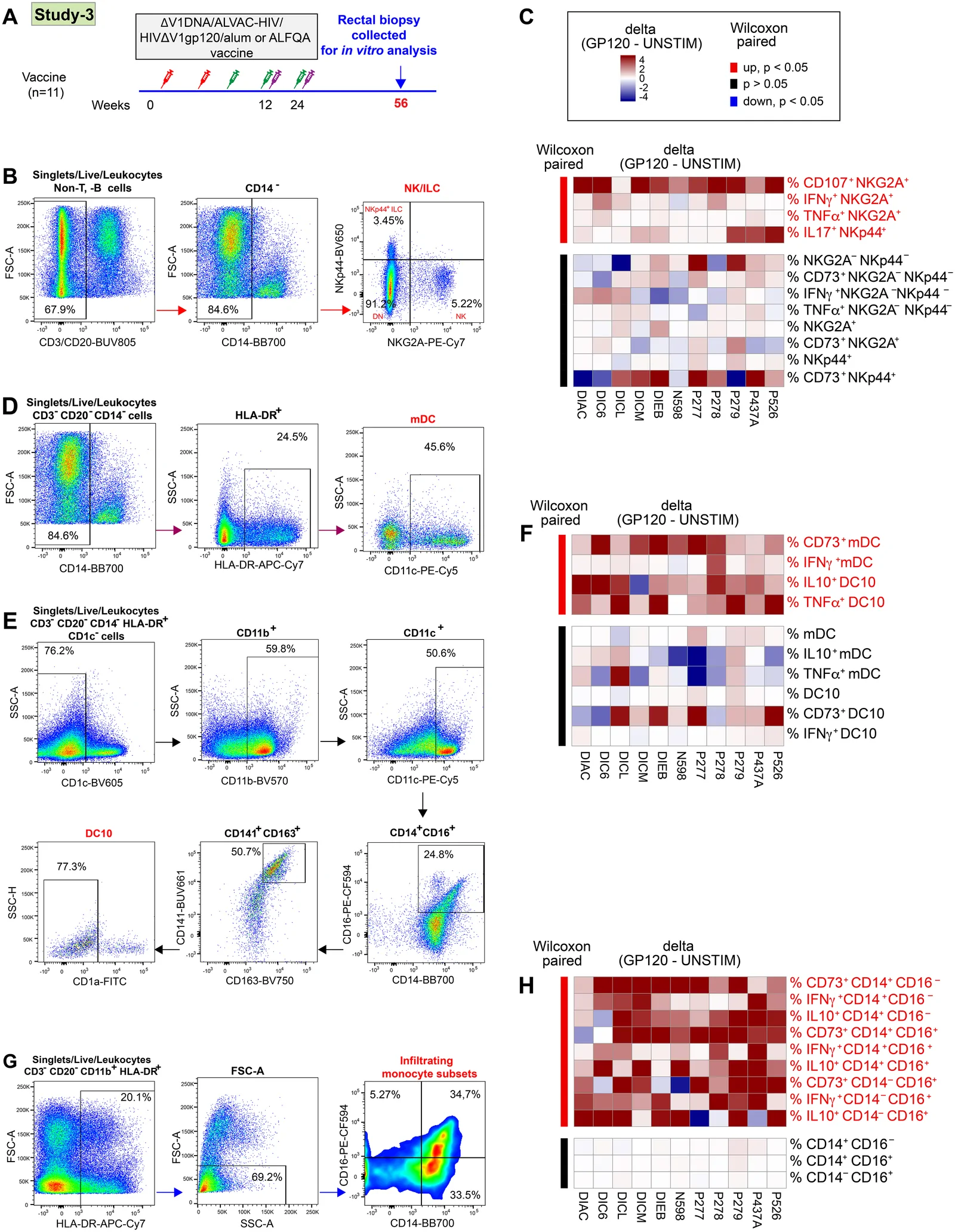
Effect of gp120 stimulation on mucosal Immune responses. **(A)** A cohort of macaques (n = 11) were primed with ΔV1 DNA-SIV at weeks 0 and 4 and boosted with ALVAC-SIV at weeks 8, 12, and 24, with or without ΔV1 gp120 in alum/ALFQA. At week 56, rectal mucosal samples were stimulated *ex vivo* with gp120 alone or gp120+SAMT-247 to evaluate *ex vivo* effects. **(B)** Gating strategy for mucosal NK/ILC subsets. **(C)** Comparison between gp120 stimulation vs unstimulated in NK/ILC responses. In the heat map, unstimulated response was subtracted from gp120 stimulated response, with paired Wilcoxon Signed Rank test p < 0.05 statistics in the left color bar. **(D, E)** Gating strategy for mDC and DC10 subsets. **(F)** Comparison between gp120 stimulation vs unstimulated in mDC and DC10 subsets responses. In the heat map, unstimulated response was subtracted from gp120 stimulated response, with paired Wilcoxon Signed Rank test p < 0.05 statistics in the left color bar. **(G)** Gating strategy for infiltrating mucosal monocytes. **(H)** Comparison between gp120 stimulation vs unstimulated in infiltrating mucosal monocytes responses. In the heat map, unstimulated response was subtracted from gp120 stimulated, with paired Wilcoxon Signed Rank test p < 0.05 statistics in the left color bar. Data in **(C, F, G)** were analyzed using two-sided paired Wilcoxon signed Rank tests.

We conducted three linked studies to evaluate SAMT-247 across exposure, *in vivo* delivery, and metabolite-specific function. In Study-1, we quantified SAMT-247 and its metabolites in vaginal tissue and vaginal secretions 4 hours after topical HEC gel administration, thereby defining the local metabolite-distribution profile following vaginal exposure (**Figure 1B**). In Study-2, we assessed sustained SAMT-247 delivery via intra vaginal ring (IVR) in animals receiving the ΔV1-SIV vaccine regimen and related plasma Met-A levels to rectal mucosal immune responses after IVR insertion (**Figure 1F**). In Study-3, we used vaccine-primed rectal mucosal cells to examine, under controlled *ex vivo* conditions, the effects of gp120 alone or in combination with SAMT-247 or individual SAMT-247 metabolites on vaccine-induced mucosal immune responses (**Figure 2A**). Together, these studies constitute a sequential exposure-to-function framework rather than a single pooled experimental cohort (**Table 1****).** Because they differed in delivery platform, sampling compartment, timing, and assay format, results were interpreted as convergent evidence for how SAMT-247-derived metabolites may shape mucosal immune responses, rather than pooled quantitatively.

**Table 1:** Summary of SAMT-247 delivery platforms, mucosal sampling compartments, and metabolite analyses across studies.

Study	SAMT-247 delivery platform	Sex of animals	Samples analyzed	SAMT-247 metabolite analysis	Rationale/limitation
Study-1	Intravaginal gel	Female	Vaginal biopsy Vaginal secretion	Metabolite bioanalysis in vaginal mucosal tissue and secretions	Designed to define local SAMT-247 metabolite distribution in the vaginal compartment after intravaginal administration.
Study-2	Continuous release by intravaginal ring	Female	Plasma and rectal biopsies	Plasma Met-A bioanalysis	Vaginal biopsies were not collected to preserve the integrity of the vaginal SIV challenge site; rectal biopsies were used for mucosal immune analysis.
Study-3	*Ex vivo* exposure to SAMT-247 or individual metabolites	Male and Female	Rectal biopsy	No paired *in vivo* metabolite analysis	Rectal tissue was used to provide a uniform mucosal sampling site across both male and female macaques for *ex vivo* immune assays.

### SAMT-247 metabolites and their distribution in blood and tissues

*In vivo*, the thioester acetyl group of SAMT-247 is rapidly transferred to endogenous thiols, including glutathione and cysteine, generating Met-B (**Figure 1A**). Met-B can subsequently undergo enzymatic methylation at the sulfur atom by thiopurine methyltransferase (TPMT), yielding Met-A, a metabolite that lacks antiviral activity (20, 26). Met-B may also undergo several alternative metabolic fates. In the presence of acetyl-CoA, it can be re-acetylated to regenerate SAMT-247 (30). Additionally, Met-B exists in equilibrium with its oxidized disulfide form, Met-C, and its benzisothiazolinone form, Met-D; both forms can revert to Met-B under reducing conditions or in the presence of excess glutathione (20, 26) (**Figure 1A**). SAMT-247, as well as its metabolites Met-C and Met-D, has been shown to inactivate HIV virions (20).

We next quantified SAMT-247 metabolites generated from degradation of the parent drug in relevant biological matrices (20, 26). To characterize *in vivo* metabolite levels, six female macaques were enrolled in Study-1 and administered 0.8% SAMT-247 HEC gel intravaginally. Four hours post-treatment, the vaginal vault was gently cleaned to remove residual gel, and vaginal swabs and biopsies were collected (**Figure 1B**). Both sample types were analyzed for SAMT-247 and its metabolites. In vaginal fluids and tissues, low levels of Met-A and higher levels of Met-D were detected (**Figures 1C–E**). Although Met-B and Met-C were readily detected in vaginal secretions, neither metabolite was quantifiable in vaginal tissue under the tissue-processing and LC-MS/MS conditions used here. The absence of quantifiable Met-B and Met-C in biopsies should be interpreted cautiously and may reflect tissue-matrix instability, processing-associated interconversion, or concentrations below the assay’s quantifiable range rather than definitive biological absence (**Figures 1C–E**). Collectively, these findings demonstrate that intravaginal administration of SAMT-247 HEC gel results in a distinct metabolite distribution profile across vaginal fluid and tissue compartments.

To further assess plasma SAMT-247 metabolite levels following intravaginal ring delivery, we performed Study-2 (24). Twelve female macaques were immunized intramuscularly with the ΔV1DNA/ALVAC-SIV/ΔV1gp120/alum regimen, consisting of SIV ΔV1gp160 and p55gag DNAs at weeks 0 and 4, ALVAC-SIV (gag-pro-gp120-TM) at weeks 8 and 12, and a ΔV1gp120 protein boost formulated in alum at week 12 in the contralateral thigh. SAMT-247-containing intravaginal rings (IVRs) were inserted 1 week after the final vaccination (week 13). Two weeks after IVR insertion, rectal mucosal biopsies were collected to assess viable-cell immune phenotyping and gp120-reactive cytokine responses, and blood samples were obtained for metabolite analysis (**Figure 1F**). Due to the limited rectal mucosal sample availability, metabolite quantification was not performed on rectal biopsies from this cohort. The plasma LC-MS/MS assay quantified Met-A (**Figure 1G**), which was the only SAMT-247-derived metabolite detectable in plasma under the assay conditions used. These data suggested systemic absorption of Met-A, however it should be interpreted as a limited systemic exposure marker and not as a direct measurement of local mucosal metabolite concentrations.

### Durable gp120-reactive rectal mucosal responses in vaccinated animals

To investigate the effects of SAMT-247 and its metabolites on mucosal immune responses *ex vivo*, we conducted Study-3. Eleven animals were immunized with the ΔV1DNA/ALVAC-HIV/ΔV1gp120/alum or ALFQA regimen, and rectal mucosal biopsies were collected 32 weeks after the final immunization (**Figure 2A**). To determine whether antigen-specific responses persisted at this time point, isolated rectal mucosal cells were stimulated *ex vivo* with gp120 and compared with unstimulated controls to assess gp120-reactive immune responses.

We first examined rectal mucosal NK/ILC populations following gp120 stimulation (**Figure 2B**) and observed gp120 induced responses in CD107a^+^ NK cells, IFN-γ^+^ NK cells, TNF-α^+^ NK cells, and IL-17^+^ NKp44^+^ ILCs (**Figure 2C**). We next evaluated dendritic cell subsets, including myeloid dendritic cells (mDCs) and DC10 cells (**Figures 2D, E**). gp120 stimulation was associated with increased frequencies of CD73^+^ mDCs, IFN-γ^+^ mDCs, IL-10^+^ DC10 cells, and TNF-α^+^ DC10 cells (**Figure 2F**). We further assessed infiltrating monocyte subsets (**Figure 2G**) and observed gp120-reactive CD14^+^CD16^-^, CD14^-^CD16^+^, and CD14^+^CD16^+^ monocyte populations expressing CD73, IL-10, or IFN-γ (**Figure 2H**).

Collectively, these findings demonstrate that gp120-reactive rectal mucosal immune responses remain detectable up to 32 weeks after the final vaccination. These results support the use of this model to evaluate the combined effects of gp120 and SAMT-247-derived metabolites to understand the antigen specific responses on mucosal immune function.

### Met-A dampens, whereas Met-D enhances, NK/ILC responses in vaccinated animals

**Figure 3:**
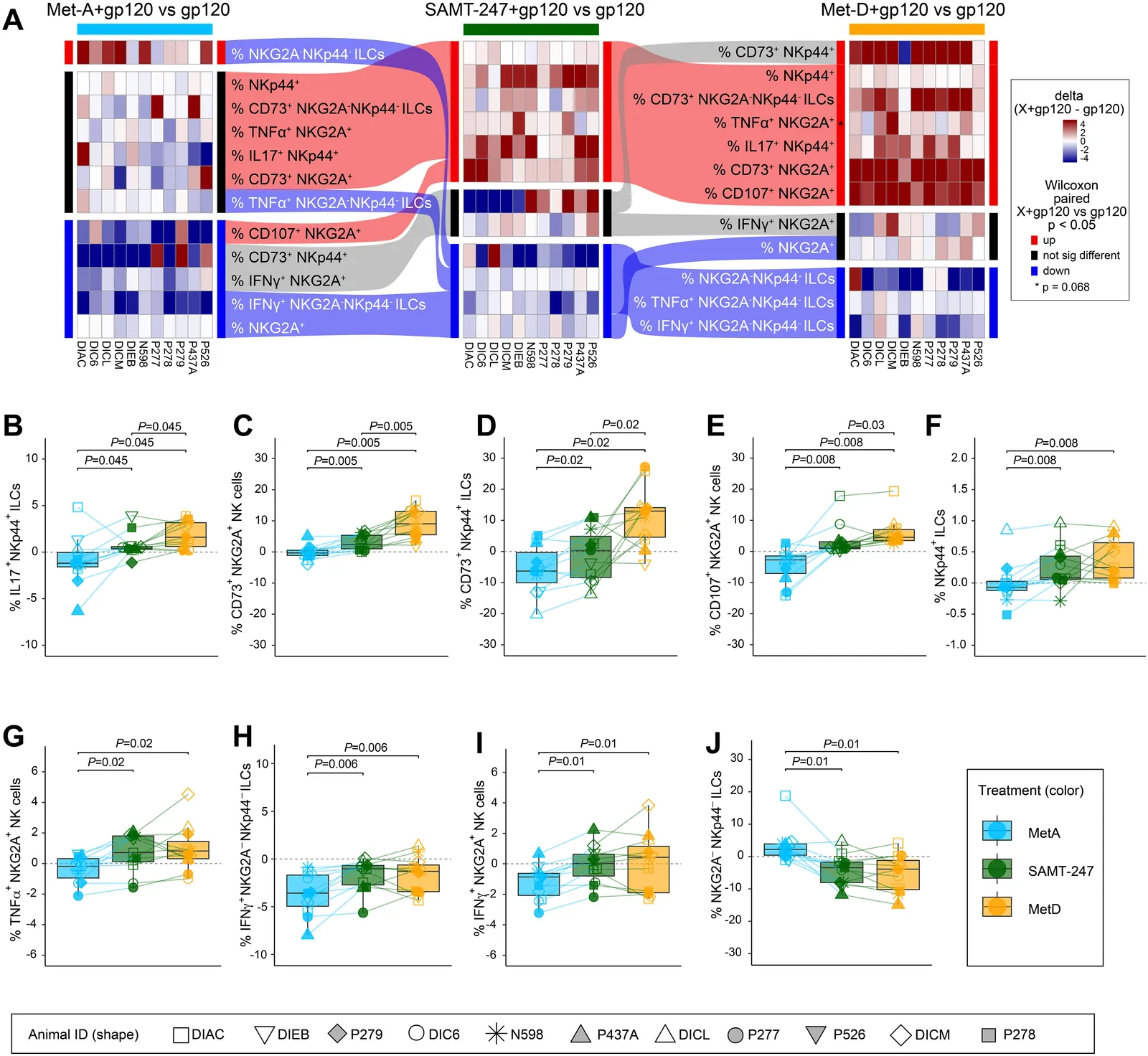
Immunomodulatory effects of SAMT-247 and its metabolites on NK/ILC responses. **(A)** Heatmaps depict stimulation-induced (gp120 response was subtracted from SAMT-247+gp120/Met-A+gp120/Met-D+gp120) changes in NK/ILC responses. Note same heat scale is used across all analytes to enable comparison of both magnitude and direction. Comparison between SAMT-247+gp120/Met-A+gp120/Met-D+gp120 stimulation vs gp120 alone for NK/ILC responses were performed. Alluvial flow connects each population across the different stimulations. **(B–J)** Effects of SAMT-247, Met-A, and Met-D on NK/ILC responses in rectal mucosa, y-axes indicate delta in percentage for each analyte (gp120 response was subtracted from SAMT-247+gp120 or Met-A+gp120 or Met-D+gp120): **(B)** IL-17^+^NKp44^+^ ILCs; **(C)** CD73^+^NKG2A^+^ NK cells; **(D)** CD73^+^ NKp44^+^ ILCs; **(E)** CD107^+^NKG2A^+^ NK cells; **(F)** NKp44^+^ ILCs; **(G)** TNF-α^+^NKG2A^+^ NK cells; **(H)** IFN-γ^+^NKG2A^+^ NK cells; **(I)** IFN-γ^+^NKG2A^-^NKp44^-^ ILCs; and **(J)** NKG2A^-^NKp44^-^ ILCs. Data were analyzed using two-sided paired Wilcoxon signed Rank tests. Box-and-whisker plots show minimum, Q1, median, Q3, and maximum values.

Cells isolated from rectal biopsies in Study-3 were stimulated *ex vivo* with gp120 alone or with gp120 in combination with SAMT-247 or one of its four metabolites (Met-A-D). Cellular phenotype and functional responses were then assessed. First, we focused on NK/ILC populations. Relative to gp120 alone, stimulation with SAMT-247+gp120, Met-B+gp120, Met-C+gp120, or Met-D+gp120 increased the frequencies of CD73^+^NKG2A^-^NKp44^-^ ILCs and CD73^+^NKG2A^+^ NK cells whereas SAMT-247+gp120, Met-C+gp120, and Met-D+gp120 increased TNF-α^+^NKG2A^+^ NK cells (**Figure 3A**; **Supplementary Figure 1A**). IL-17^+^NKp44^+^ ILCs, total NKp44^+^ ILCs, and CD107a^+^NKG2A^+^ NK cells were significantly elevated following SAMT-247+gp120 and Met-D+gp120 stimulation, while CD73^+^NKp44^+^ ILCs increased after Met-B+gp120, Met-C+gp120, and Met-D+gp120 treatment (**Figure 3A**; **Supplementary Figure 1A**). In contrast, Met-A+gp120 did not enhance these populations, and both Met-A+gp120 and Met-B+gp120 reduced CD107a^+^NKG2A^+^ NK cell responses (**Figure 3A**; **Supplementary Figure 1A**). Met-B+gp120 and Met-C+gp120 also decreased NKp44^+^ ILC responses (**Supplementary Figure 1A**). Conversely, IFN-γ^+^NKG2A^-^NKp44^-^ ILCs were reduced following SAMT-247+gp120 and all metabolite+gp120 stimulations. Total NKG2A^-^NKp44^-^ ILCs were decreased by SAMT-247+gp120, Met-B+gp120, Met-C+gp120, and Met-D+gp120, but were increased by Met-A+gp120. NKG2A^+^ NK cells were reduced after SAMT-247+gp120, Met-A+gp120, and Met-B+gp120 stimulation, whereas TNF-α^+^NKG2A^-^NKp44^-^ ILCs were decreased by SAMT-247+gp120, Met-C+gp120, and Met-D+gp120 (**Figure 3A**; **Supplementary Figure 1A**). Overall, the response profile induced by Met-D+gp120 most closely resembled that induced by SAMT-247+gp120, whereas Met-A+gp120 produced a distinct and generally attenuated pattern. Met-B+gp120 and Met-C+gp120 elicited overlapping NK/ILC responses that partially resembled those observed with SAMT-247+gp120 (**Figure 3A**; **Supplementary Figure 1A**).

Direct comparison with SAMT-247 further highlighted the divergent effects of Met-A and Met-D. Relative to SAMT-247, Met-A failed to increase IL-17^+^NKp44^+^ ILCs (*P* = 0.045; **Figure 3B**), CD73^+^NKG2A^+^ NK cells (*P* = 0.005; **Figure 3C**), reduced CD73^+^NKp44^+^ ILCs (*P* = 0.02; **Figure 3D**) and CD107^+^NKG2A^+^ NK cells (*P* = 0.008; **Figure 3E**). In contrast, Met-D stimulation increased all of these populations relative to SAMT-247 induction (*P* = 0.045, *P* = 0.005, *P* = 0.02 and *P* = 0.03, respectively; **Figures 3B–E**). Met-A further failed to increase NKp44^+^ ILCs (*P* = 0.008; **Figure 3F**) and TNF-α^+^NKG2A^+^ NK cells (*P* = 0.02; **Figure 3G**). Met-A also decreased IFN-γ^+^NKG2A^-^NKp44^-^ ILCs (*P* = 0.006; **Figure 3H**) and IFN-γ^+^NKG2A^+^ NK cells (*P* = 0.01; **Figure 3I**) more than SAMT-247, while increasing total NKG2A^-^NKp44^-^ ILCs despite the decreases observed with SAMT-247 and Met-D (*P* = 0.01 for both comparisons; **Figure 3J**). In contrast, Met-D closely recapitulated the SAMT-247 response profile across these NK/ILC subsets (**Figures 3F–J**).

Compared with SAMT-247, both Met-B and Met-C increased CD73^+^NKp44^+^ ILCs (*P* = 0.02 for both; **Supplementary Figure 1B**), CD73^+^NKG2A^+^ NK cells (*P* = 0.01 for both; **Supplementary Figure 1C**), and CD73^+^NKG2A^-^NKp44^-^ ILCs (*P* = 0.01 for both; **Supplementary Figure 1D**), consistent with the effects of Met-D on selected CD73^+^ populations (**Figures 3C, D**). However, similar to Met-A (**Figure 3D**), both Met-B and Met-C reduced CD107a^+^NKG2A^+^ NK cells (*P* = 0.03 for both; **Supplementary Figure 1E**), rather than reproducing the increase observed after SAMT-247 and Met-D stimulations. In addition, Met-C significantly decreased total NKp44^+^ ILCs, while Met-B showed a similar trend (*P* = 0.01 and *P* = 0.06, respectively; **Supplementary Figure 1F**). Finally, although Met-D and SAMT-247 produced comparable decreases in total NKG2A^-^NKp44^-^ ILCs (**Figure 3J**), both Met-B and Met-C induced more pronounced reductions in this population (*P* = 0.04 for both; **Supplementary Figure 1G**).

Taken together, these findings indicate that Met-D largely recapitulates the immunomodulatory effects of SAMT-247 on NK/ILC populations, whereas Met-A fails to reproduce and, in some cases, opposes the SAMT-247-associated immune profile. Met-B and Met-C mirrored selected SAMT-247-mediated effects in a subset-dependent manner. Overall, Met-A broadly dampened NK/ILC responses, whereas Met-B, Met-C, and especially Met-D preserved SAMT-247-like immune signatures. Notably, Met-D uniquely enhanced IL-17^+^NKp44^+^ ILC and CD107a^+^NKG2A^+^ NK cell responses relative to the other metabolites, a pattern consistent with stronger mucosal barrier-associated immunity (7, 24, 27–29, 31) and greater ADCC linked (6, 7, 24, 27, 31–33) antiviral effector potential.

### SAMT-247, Met-B, Met-C, and Met-D promote anti-inflammatory mucosal immunity through induction of tolerogenic DC10 cells

**Figure 4:**
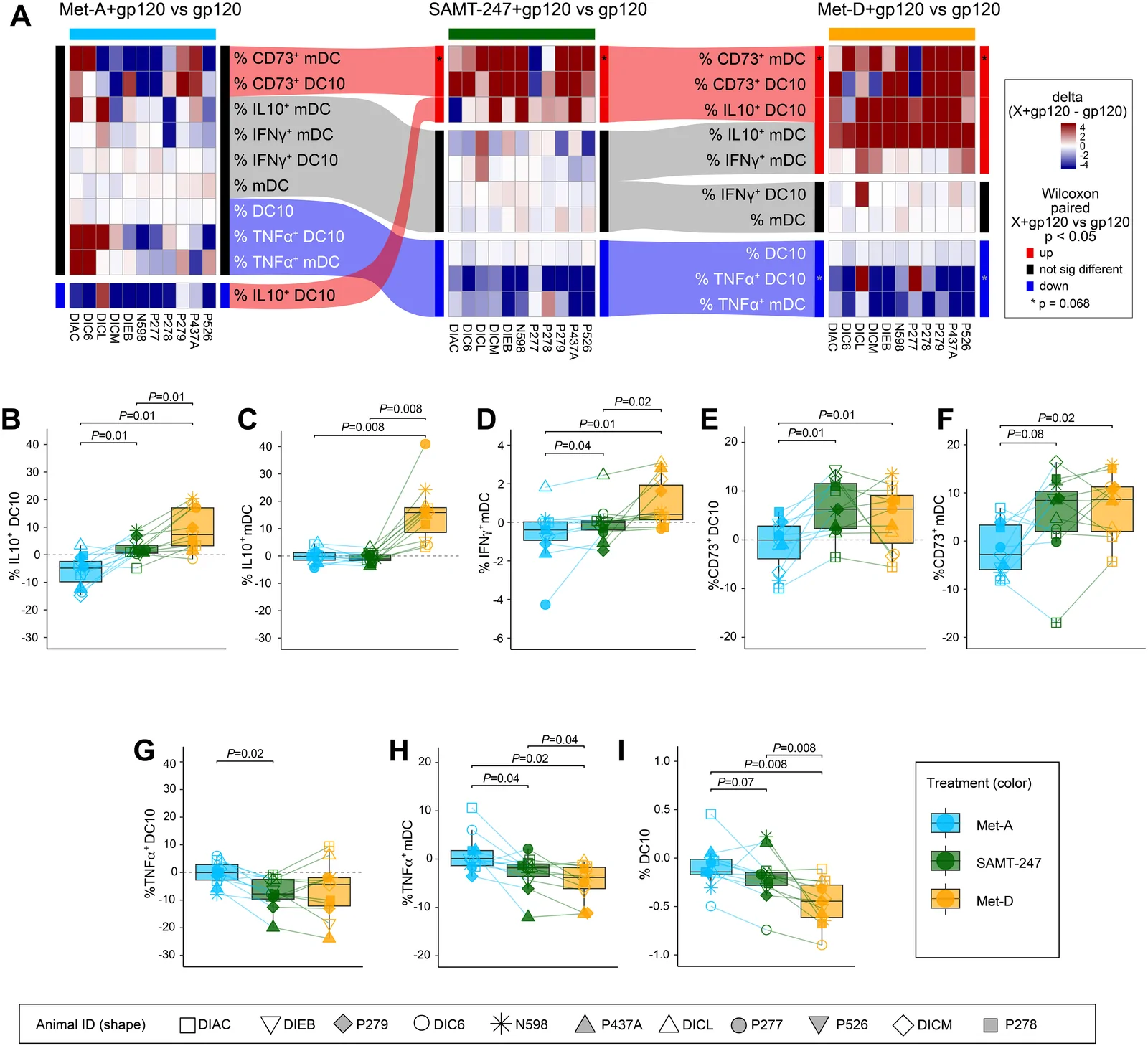
Differential effects of Met-A and Met-D on mucosal dendritic cells. **(A)** Heatmaps depict stimulation-induced (gp120 response was subtracted from SAMT-247+gp120/Met-A+gp120/Met-D+gp120) changes in DC10 and mDC responses. Note same heat scale is used across all analytes to enable comparison of both magnitude and direction. Comparison between SAMT-247+gp120/Met-A+gp120/Met-D+gp120 stimulation vs gp120 alone for DC responses were performed. Alluvial flow connects each population across the different stimulation. **(B–I)** Effects of SAMT-247, Met-A, and Met-D on dendritic cell responses, y-axes indicate delta in percentage for each analyte (gp120 response was subtracted from SAMT-247+gp120 or Met-A+gp120 or Met-D+gp120): **(B)** IL-10^+^ DC10; **(C)** IL-10^+^ mDC; **(D)** IFN-γ^+^ mDC; **(E)** CD73^+^ DC10; **(F)** CD73^+^ mDC; **(G)** TNF-α^+^ DC10; **(H)** TNF-α^+^ mDC; and **(I)** DC10. Data were analyzed using two-sided paired Wilcoxon signed Rank tests. Box-and-whisker plots show minimum, Q1, median, Q3, and maximum values.

We next examined the effects of SAMT-247 and its metabolites on dendritic cell populations. Relative to gp120 alone, stimulation with SAMT-247+gp120, Met-B+gp120, Met-C+gp120, and Met-D+gp120 each significantly increased the frequencies of CD73^+^ mDCs, CD73^+^ DC10 cells, and IL-10^+^ DC10 cells (**Figure 4A**, **Supplementary Figure 2A**). Compared with SAMT-247, the induction of IL-10^+^ DC10 cells was greater following Met-B and Met-C stimulation (*P* = 0.01 for both; **Supplementary Figure 2B**) and Met-D stimulation (*P* = 0.01; **Figure 4B**), whereas Met-B showed a trend toward a greater increase in CD73^+^ DC10 cells (*P* = 0.06; **Supplementary Figure 2C**). Met-B, Met-C, and Met-D also increased IL-10^+^ mDC frequencies, whereas SAMT-247 did not measurably alter this population (Met-B and Met-C, *P* = 0.01 for both; **Supplementary Figure 2D**; Met-D, *P* = 0.008; **Figure 4C**). In addition, Met-D increased IFN-γ^+^ mDCs relative to the absence of change with SAMT-247 (*P* = 0.02; **Figure 4D**), and Met-B+gp120 increased IFN-γ^+^ DC10 cells relative to gp120 alone (**Supplementary Figure 2A**). In contrast, Met-A+gp120 did not enhance these dendritic cell subsets relative to gp120 alone (**Figure 4A**) and was associated with reduced IL-10^+^ DC10 responses compared with SAMT-247 (*P* = 0.01; **Figure 4B**).

Conversely, total DC10 cells, TNF-α^+^ DC10 cells, and TNF-α^+^ mDCs were all reduced following stimulation with SAMT-247+gp120, Met-B+gp120, Met-C+gp120, or Met-D+gp120 relative to gp120 alone, whereas Met-A+gp120 had no significant effect on these populations (**Figure 4A**, **Supplementary Figure 2A**). Direct comparison with SAMT-247 further underscored the distinct profile of Met-A. Specifically, Met-A significantly reduced IFN-γ^+^ mDCs (*P* = 0.04; **Figure 4D**), failed to increase CD73^+^ DC10 cells (*P* = 0.01; **Figure 4E**) and CD73^+^ mDCs (*P* = 0.02; **Figure 4F**), and failed to reduce TNF-α^+^ DC10 cells (*P* = 0.02; **Figure 4G**) and TNF-α^+^ mDCs (*P* = 0.04; **Figure 4H**), with a similar trend observed for total DC10 cells (*P* = 0.07; **Figure 4I**). By contrast, Met-C and Met-D produced greater reductions in total DC10 cells (*P* = 0.01; **Supplementary Figure 2E**, *P* = 0.008; **Figure 4I**) and TNF-α^+^ mDCs (*P* = 0.04; **Supplementary Figure 2F**, *P* = 0.04; **Figure 4H**) than SAMT-247. Collectively, these findings indicate that Met-B, Met-C, and Met-D largely recapitulate, and in some settings enhance, the SAMT-247-associated immunomodulatory profile by promoting tolerogenic and anti-inflammatory dendritic cell responses, whereas Met-A broadly fails to regulate DC function in this manner and may instead favor inflammatory skewing.

### Met-A favors pro-inflammatory monocyte activation, whereas Met-B, Met-C, and Met-D promote an anti-inflammatory mucosal profile

**Figure 5:**
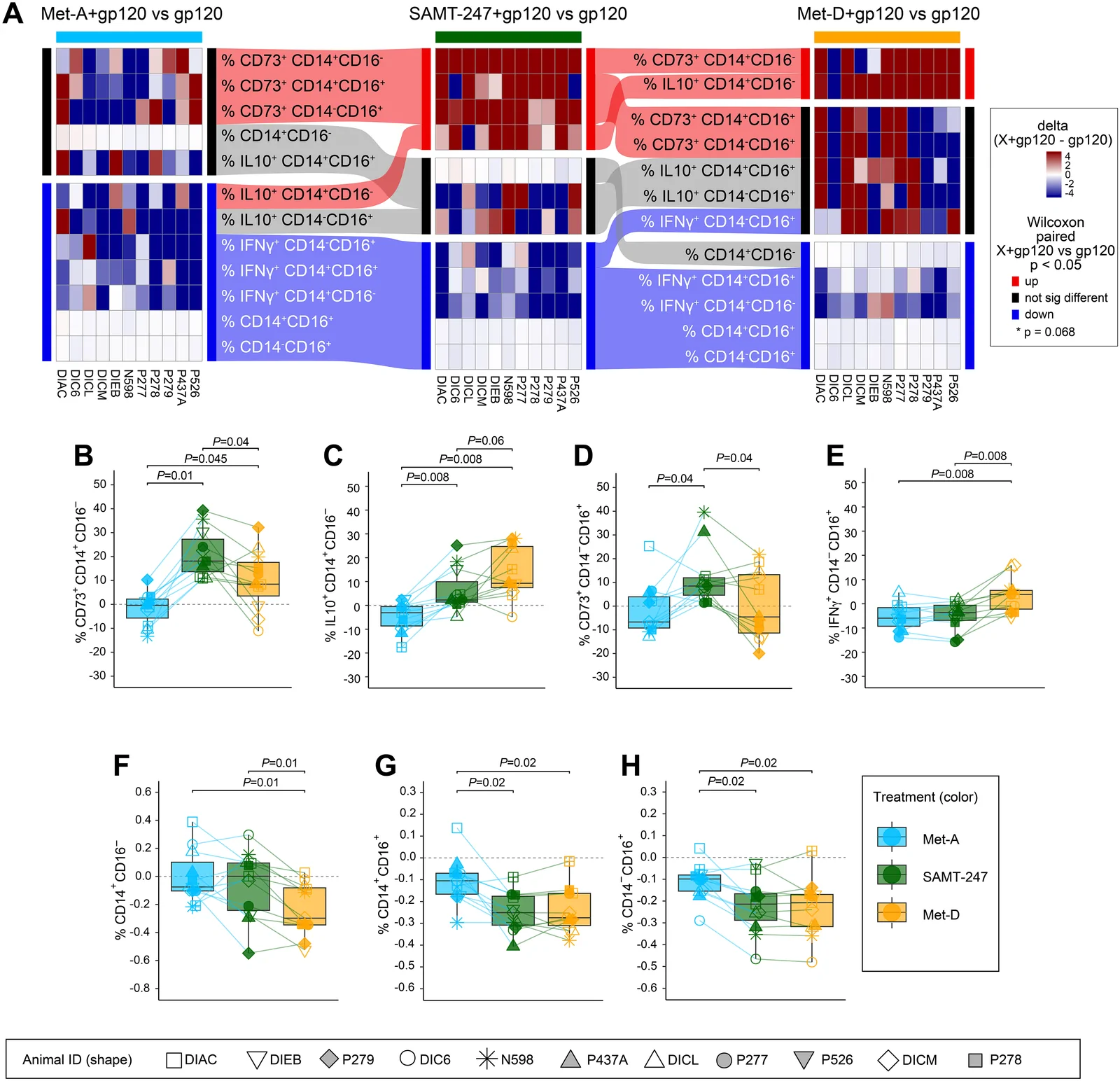
Met-A induces pro-inflammatory and Met-D anti-inflammatory mucosal monocyte responses. **(A)** Heatmaps depict stimulation-induced (gp120 response was subtracted from SAMT-247+gp120/Met-A+gp120/Met-D+gp120) changes in infiltrating monocyte responses. Note same heat scale is used across all analytes to enable comparison of both magnitude and direction. Comparison between SAMT-247+gp120/Met-A+gp120/Met-D+gp120 stimulation vs gp120 alone for monocyte responses were performed. Alluvial flow connects each population across the different stimulation. **(B–H)** Effects of SAMT-247, Met-A, and Met-D on monocyte subsets, y-axes indicate delta in percentage for each analyte (gp120 response was subtracted from SAMT-247+gp120 or Met-A+gp120 or Met-D+gp120): **(B)** CD73^+^ CD14^+^CD16^-^; **(C)** IL-10^+^ CD14^+^CD16^-^; **(D)** CD73^+^ CD14^-^CD16^+^; **(E)** IFN-γ^+^ CD14^-^CD16^+^; **(F)** CD14^+^CD16^-^; **(G)** CD14^+^CD16^-^; and **(H)** CD14^-^CD16^+^. Data were analyzed using two-sided paired Wilcoxon signed Rank tests. Box-and-whisker plots show minimum, Q1, median, Q3, and maximum values.

We next evaluated infiltrating rectal mucosal monocyte subsets. Relative to gp120 alone, stimulation with SAMT-247+gp120, Met-B+gp120, Met-C+gp120, and Met-D+gp120 each increased the frequencies of CD73^+^CD14^+^ and IL-10^+^CD14^+^ monocytes (**Figure 5A**, **Supplementary Figure 3A**). Compared with SAMT-247, the induction of CD73^+^CD14^+^ monocytes were less dramatic with Met-D (*P* = 0.04; **Figure 5B**), whereas Met-B showed a trend toward greater induction (*P* = 0.06; **Supplementary Figure 3B**). Similarly, IL-10^+^CD14^+^ monocytes were more strongly induced by Met-B and Met-C (*P* = 0.008 for both; **Supplementary Figure 3C**), with a similar trend observed for Met-D (*P* = 0.06; **Figure 5C**), relative to SAMT-247. In contrast, Met-A+gp120 did not alter either subset relative to gp120 alone (**Figure 5A**) and therefore failed to reproduce the SAMT-247-associated increases in CD73^+^CD14^+^ and IL-10^+^CD14^+^ monocytes (*P* = 0.01 and *P* = 0.008 respectively, **Figures 5B, C**).

Stimulation with SAMT-247+gp120, Met-B+gp120, and Met-C+gp120 also increased CD73^+^CD14^+^CD16^+^ monocytes (**Supplementary Figure 3A**), whereas SAMT-247+gp120 and Met-B+gp120 increased CD73^+^CD16^+^ monocytes relative to gp120 alone (**Supplementary Figure 3A**). Neither subset was altered by Met-A+gp120 or Met-D+gp120 (**Figure 5A**), and accordingly both Met-A and Met-D failed to increase CD73^+^CD16^+^ monocytes relative to SAMT-247 (*P* = 0.04 for both; **Figure 5D**). IL-10^+^CD14^+^CD16^+^ monocytes remained unchanged following SAMT-247+gp120 or any metabolite treatment (**Figure 5A**, **Supplementary Figure 3A**). By contrast, Met-A+gp120 and Met-B+gp120 reduced IL-10^+^CD16^+^ monocytes, whereas this population was unchanged after SAMT-247+gp120, Met-C+gp120, and Met-D+gp120 stimulation (**Figure 5A**, **Supplementary Figure 3A**). Relative to the reductions observed with SAMT-247+gp120, Met-A+gp120, and Met-B+gp120 (**Figure 5A**, **Supplementary Figure 3A**), Met-D (*P* = 0.008; **Figure 5E**) and Met-C (*P* = 0.04, **Supplementary Figure 3D**) failed to reduce IFN-γ^+^CD16^+^ monocytes. All five stimulation conditions reduced IFN-γ^+^CD14^+^CD16^+^, IFN-γ^+^CD14^+^, and total CD14^+^CD16^+^ monocyte populations relative to gp120 alone (**Figure 5A**, **Supplementary Figure 3A**). A reduction in total CD14^+^ monocytes was only evident following Met-D+gp120 stimulation and differed significantly from the lack of SAMT-247 response (**Figure 5A**, **Supplementary Figure 3A**, *P* = 0.01 relative to SAMT-247; **Figure 5F**). However, the decreases in total CD14^+^CD16^+^ and total CD14^+^ monocytes were less pronounced with Met-A than with SAMT-247 (*P* = 0.02 for both; **Figures 5G, H**).

Taken together, these findings indicate that, although depletion of IFN-γ^+^ monocyte subsets was a common feature across conditions, SAMT-247 metabolites exert distinct effects on infiltrating rectal mucosal monocytes. Met-A was associated with a more pro-inflammatory profile, characterized by failure to induce CD73-expressing monocytes, reduced IL-10-producing monocyte responses, and less dramatic depletion of total CD14^+^CD16^+^ and CD14^+^ monocyte populations. In contrast, Met-B, Met-C, and Met-D favored a more anti-inflammatory mucosal profile, particularly through enhanced induction of IL-10^+^CD14^+^ monocytes.

### Exploratory associations between plasma Met-A and rectal mucosal immune responses

**Figure 6:**
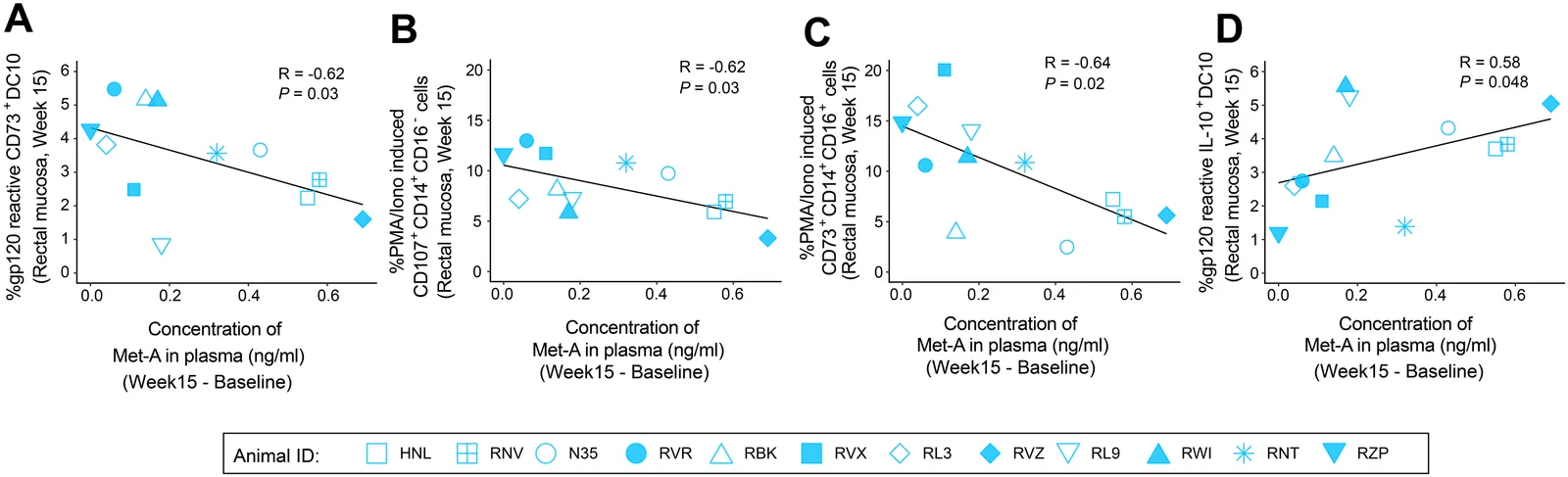
Correlation analysis of plasma Met-A with mucosal immune responses. **(A–D)**. Correlation of concentration of Met-A in plasma (n=12) with **(A)** gp120-reactive CD73^+^DC10; **(B)** PMA/Ionomycin induced CD107^+^CD14^+^CD16^-^ cells; **(C)** PMA/Ionomycin induced CD73^+^CD14^+^CD16^+^ cells; and **(D)** gp120-reactive IL10^+^ DC10. Data were analyzed using two-sided Spearman correlation. Correlations were exploratory; P values are nominal and were not adjusted for multiple comparisons.

We performed correlation analyses in ΔV1DNA/ALVAC-SIV/ΔV1gp120/alum-vaccinated animals treated with SAMT-247 in Study-2 to examine the relationship between plasma Met-A concentrations and rectal mucosal immune responses (**Figure 1F**; **Supplementary Figure 4**). gp120-reactive CD73^+^ DC10 cells in the mucosa were negatively correlated with plasma Met-A concentrations (*P* = 0.04, R= -0.62; **Figure 6A**), consistent with our *ex vivo* observation that Met-A failed to increase CD73^+^ DC10 frequencies relative to SAMT-247 (**Figure 4E**). In addition, PMA/ionomycin-induced CD107a^+^CD14^+^CD16^-^ cells (*P* = 0.04, R= -0.62; **Figure 6B**) and CD73^+^CD14^+^CD16^+^ monocytes (*P* = 0.03, R= -0.64; **Figure 6C**) were also negatively correlated with plasma Met-A concentrations. Although CD107a expression in CD14^+^CD16^-^ cells was not directly assessed *ex vivo*, Met-A reduced CD107a expression in NKG2A^+^ NK cells (**Figure 3E**). Similarly, Met-A failed to increase CD73 expression on CD14^+^CD16^-^ and CD14^+^CD16^+^ monocytes relative to SAMT-247 stimulation *ex vivo* (**Figures 5B, D**), suggesting modest concordance between the *ex vivo* and *in vivo* findings.

In contrast, gp120-reactive IL-10^+^ DC10 cells were positively associated with plasma Met-A concentrations (*P* = 0.048, R = 0.58; **Figure 6D**), whereas Met-A reduced IL-10^+^ DC10 cells *ex vivo* (**Figure 4B**), indicating a discordant relationship for this subset.

Together, these exploratory analyses indicate that plasma Met-A was associated with selected rectal mucosal immune responses, but plasma Met-A should not be interpreted as a direct surrogate for local mucosal metabolite exposure.

### Summary of metabolite effects on immune signatures previously linked to reduced SIV acquisition risk

**Figure 7:**
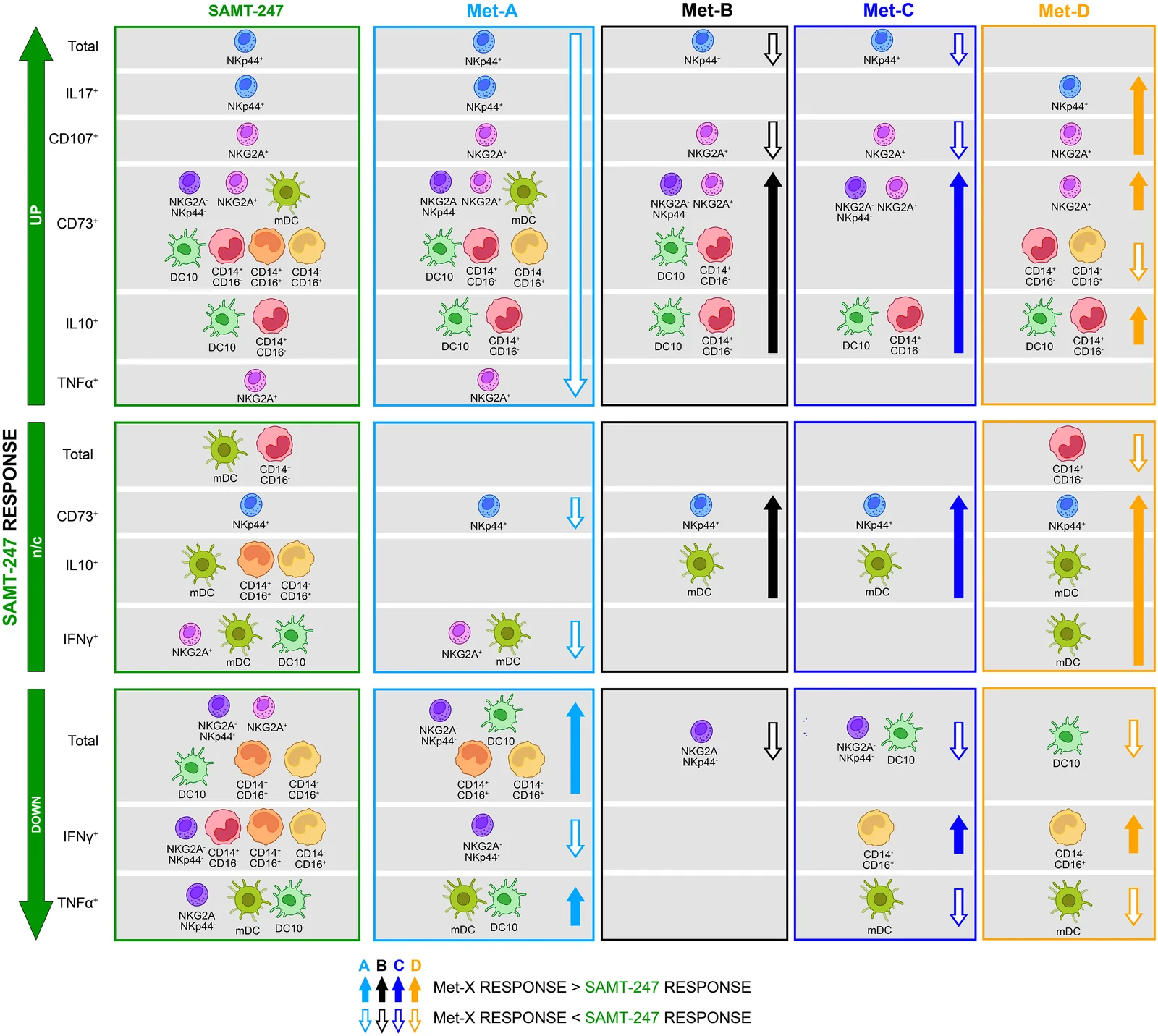
SAMT-247 metabolites differentially shape mucosal innate immune responses. Schematic summary of mucosal immune responses induced by SAMT-247 and its metabolites in *ex vivo* gp120-stimulated mucosal cells from vaccinated macaques. Immune populations are grouped according to whether they were increased or decreased by SAMT-247 relative to gp120 alone. The upper panel summarizes cell populations increased by SAMT-247, including NKp44^+^ innate lymphoid cells (ILCs), IL-17^+^NKp44^+^ ILCs, CD107a^+^NKG2A^+^ natural killer (NK) cells, CD73^+^ NK/ILC subsets, CD73^+^ myeloid dendritic cells (mDCs), CD73^+^ DC10 cells, CD73^+^ monocyte subsets, IL-10^+^ DC10 or monocyte populations and TNF-α^+^ NKG2A^+^ NK cells. The middle panel summarizes populations unchanged in SAMT-247, including total mDC and CD14^+^ monocytes, CD73^+^ NKp44^+^ ILCs, IL-10^+^ mDC, CD14^+^CD16^+^ and CD14^-^CD16^+^ monocytes, IFN- γ^+^ NKG2A^+^ NK, and dendritic cells. The lower panel summarizes populations reduced by SAMT-247, including selected total immune, IFN-γ^+^ and TNF-α^+^ NK/ILC, dendritic cell, and monocyte subsets. Each metabolite panel illustrates the effect of Met-A, Met-B, Met-C, or Met-D relative to these SAMT-247-associated responses. Filled upward arrows indicate responses greater than those induced by SAMT-247, whereas open downward arrows indicate responses lower than those induced by SAMT-247. n/c = not changed SAMT-247+gp120 vs gp120. Met-D most closely recapitulates and strengthens the SAMT-247-associated protective profile by enhancing IL-17^+^NKp44^+^ ILCs, CD107a^+^NKG2A^+^ NK cells, and regulatory CD73^+^ or IL-10^+^ myeloid populations while limiting inflammatory subsets. Met-B and Met-C partially reproduce this profile, particularly through induction of CD73^+^ and IL-10^+^ myeloid responses but show weaker effects on antiviral NK/ILC populations. In contrast, Met-A fails to sustain several protective SAMT-247-associated responses and is associated with reduced regulatory and cytolytic signatures, consistent with inflammatory skewing. The cell pictures were obtained from Biorender, further modified using Affinity design

This analysis was designed to contextualize the *ex vivo* effects of SAMT-247 metabolites in the context of immune signatures previously associated with reduced SIV acquisition risk, rather than to define new correlates of protection. Within this framework, SAMT-247 and its metabolites differentially modulated several innate immune populations that have been linked to protection in ΔV1DNA/ALVAC-SIV/ΔV1gp120/alum-vaccinated macaques. Protective NKp44^+^ and IL-17^+^NKp44^+^ ILC subsets (7, 24, 27–29, 31) were increased by SAMT-247 and were similarly enhanced by Met-D. In contrast, Met-A failed to increase these responses, whereas Met-B and Met-C reduced NKp44^+^ ILC frequencies (**Figure 7**). CD107a^+^NKG2A^+^ NK cells, which may contribute to ADCC, another correlate of protection (6, 7, 24, 27, 31–34), were likewise increased by SAMT-247 and Met-D, while Met-A and Met-B attenuated this response (**Figure 7**).

Protective myeloid populations, including DC10 cells (6, 24, 35)and infiltrating monocytes (6, 7, 24, 27, 35), also exhibited a regulatory shift. Although total DC10 frequencies were reduced by SAMT-247, Met-B, Met-C, and Met-D, the frequencies of IL-10^+^ and CD73^+^ DC10 cells were increased. CD73 (NT5E), an ecto-5′-nucleotidase that generates immunosuppressive adenosine (36), was also upregulated on mDCs, CD14^+^ infiltrating monocytes, NKG2A^-^NKp44^-^ and NKG2A^+^ NK/ILC subsets following stimulation with SAMT-247, Met-B, Met-C, and Met-D. By contrast, Met-A reduced CD73^+^ NKp44^+^ ILCs and failed to alter other CD73-expressing immune populations, consistent with loss of a regulatory mucosal environment that may otherwise limit HIV susceptibility (**Figure 7**).

Importantly, inflammatory populations associated with susceptibility, including NKG2A^-^NKp44^-^ ILCs (27–29, 31), TNF-α^+^ DC10 cells, and TNF-α^+^ mDCs, were reduced by SAMT-247 and remained low or were further decreased following exposure to Met-B, Met-C, and Met-D. In contrast, Met-A either increased these inflammatory signatures or failed to suppress them (**Figure 7**).

Taken together, these findings indicate that SAMT-247 establishes a balanced mucosal immune profile characterized by both regulatory and antiviral features. Among its metabolites, Met-D most closely recapitulates and, in some respects, strengthens this protective signature by enhancing IL-17^+^NKp44^+^ ILC and ADCC-associated NK responses while preserving regulatory CD73^+^ and IL-10^+^ myeloid subsets. Met-B and Met-C partially reproduce these effects, whereas Met-A disrupts this balance and promotes inflammatory skewing.

## Discussion

In this study, we demonstrate a differential distribution of SAMT-247 metabolites within the female genital tract of rhesus macaques. Vaginal fluids contained low levels of Met-A and higher levels of Met-B, Met-C, and Met-D, whereas in vaginal tissues we observed low Met-A and enrichment of Met-D. Functional analyses demonstrated that these metabolites exert distinct immunologic effects. Met-A promoted an inflammation-associated mucosal profile, whereas Met-D reinforced immune signatures previously associated with protection against SIV_mac251_ acquisition in the ΔV1DNA/ALVAC-SIV/ΔV1gp120/alum vaccine model (7, 24, 27, 32, 33, 35). Given the role of mucosal inflammation in target-cell recruitment and activation, these distinct profiles may have direct implications for HIV/SIV acquisition risk.

SAMT-247, when combined with the ΔV1DNA/ALVAC-SIV/ΔV1gp120/alum vaccine regimen, shapes a mucosal immune environment strongly associated with protection against SIV_mac251_ acquisition. Consistent with prior correlates of protection, SAMT-247 enhanced IL-17^+^NKp44^+^ ILCs (7, 24, 27–29, 31), CD107a^+^NKG2A^+^ NK cells with protective ADCC potential (31) and CD73^+^ cell populations (24, 35), while reducing inflammatory IFN-γ^+^NKG2A^-^NKp44^-^ ILCs (27–29, 31) and several TNF-α producing dendritic cell subsets. Met-D closely mirrored, and in some cases strengthened, this profile. It increased IL-17^+^NKp44^+^ ILCs, augmented CD107a^+^NKG2A^+^ NK cells with ADCC potential, and expanded IL-10^+^ DC10 and CD14^+^ monocytes, while also supporting CD73^+^ regulatory monocyte populations. At the same time, Met-D maintained or reduced inflammatory DC and ILC subsets and decreased total CD14^+^ monocytes, suggesting controlled immune activation. Together, these features indicate preservation of epithelial-supportive ILC3-like responses (37, 38), enhanced cytolytic NK function, and reinforcement of regulatory myeloid networks.

Met-B and Met-C partially recapitulated this protective immunological landscape. Both increased IL-10^+^ DC10 and IL-10^+^ CD14^+^ monocytes and Met-B further enhanced CD73^+^ regulatory subsets. However, their effects on NK were more limited, and Met-B was associated with a modest increase in IFN-γ^+^ DC10, indicating selective rather than comprehensive preservation of vaccine-associated innate signatures.

In contrast, Met-A consistently shifted the mucosal environment toward inflammation. It failed to increase IL-17^+^NKp44^+^ ILCs and CD73^+^ populations, while diminishing IL-10^+^ dendritic cell and monocyte subsets, and failing to decrease TNF-α producing DCs and inflammatory CD14^+^CD16^+^ monocytes. These findings suggest that Met-A is not merely virologically inactive (20, 25, 26); it also fails to recapitulate the SAMT-247 associated regulatory and antiviral profile and may instead promote inflammatory skewing.

The newly developed plasma bioanalytical method enabled quantification of Met-A. Extending this approach to Met-D remains analytically challenging because SAMT-247-related sulfanylbenzamide thioesters are chemically labile and susceptible to hydrolysis, nucleophilic attack, and blood-associated metabolism (39). Prior work has also shown that related sulfanylbenzamide species can be rapidly metabolized in blood and may partition preferentially into whole blood rather than plasma, consistent with interaction with blood cellular components (40). Therefore, plasma metabolite measurements may underestimate or incompletely reflect total SAMT-247-related exposure, particularly for Met-D. The absence of plasma Met-D measurements should therefore not be interpreted as evidence that Met-D was absent *in vivo* or that prolonged IVR exposure shifted SAMT-247 metabolism toward Met-A. Instead, this finding may reflect matrix- and compartment-specific differences in metabolite stability, distribution, and assay performance. Future studies will require optimized and validated assays for Met-D and other metabolites in plasma, and relevant mucosal compartments.

In Study 2, plasma Met-A concentrations were correlated with rectal mucosal innate immune-cell populations in vaccinated macaques receiving SAMT-247-releasing IVRs. These hypothesis-generating analyses focused on plasma Met-A and these data should be interpreted as associations between measurable systemic Met-A exposure and rectal mucosal immune responses, rather than as direct evidence of Met-A-mediated immune modulation. The rectal immune-response data from this study have been reported separately by Rahman et al. (24). Future studies directly quantifying Met-D in systemic and mucosal compartments, together with paired immune-response analyses, will help determine the extent to which *in vivo* observations align with the current *ex vivo* findings. Higher plasma Met-A levels were inversely correlated with gp120-reactive CD73^+^ DC10 cells, PMA/ionomycin-induced CD107^+^CD14^+^CD16^-^ cells, and PMA/ionomycin-induced CD73^+^CD14^+^CD16^+^ cells. The *ex vivo* panel evaluated these markers on related rather than identical cell populations, and these populations showed patterns generally consistent with the correlation analysis. In contrast, positive association between plasma Met-A and gp120-reactive IL-10^+^ DC10 cells differed from the *ex vivo* response, highlighting the context dependence of metabolite-immune relationships across experimental systems. In particular, plasma Met-A may not accurately represent local mucosal Met-A exposure. Furthermore, the effects of SAMT-247 and its metabolites on immune cells were tested individually *ex vivo* (Study-3), whereas IVR delivery *in vivo* resulted in concurrent exposure to multiple drug components within an intact vaccinated mucosal environment (Study-2). Thus, the *ex vivo* data partially support the *in vivo* observations, while also highlighting the greater complexity of drug-component exposure *in vivo*.

Although the vaginal and rectal mucosa are both relevant sites of mucosal SIV/HIV exposure, they are anatomically and immunologically distinct compartments. In macaques, the vaginal mucosa is lined by stratified squamous epithelium, with susceptible CD4^+^CCR5^+^ T cells, macrophages, and dendritic cells located primarily in the lamina propria. In contrast, the rectal mucosa is lined by a single-layer columnar epithelium and contains abundant lamina propria immune cells positioned closer to the lumen, including DC-SIGN^+^ dendritic cells and CCR5^+^CD4^+^DC-SIGN^+^ cells (41–43). Consistent with these compartmental differences, early SIV target-cell populations may vary by route of exposure, with Th17-lineage CD4^+^ T cells preferentially infected after vaginal challenge and both Th17 cells and immature dendritic cells identified as early targets after rectal challenge (44, 45). The study was designed to characterize vaginal SAMT-247 metabolite distribution and assess the effects of individual drug components on mucosal immune cells. In Study-1, following intravaginal SAMT-247 gel administration, vaginal tissue was collected for SAMT-247 bioanalysis. In Study-2, to preserve the integrity of the vaginal SIV challenge site, rectal mucosa was selected as a complementary immune-sampling compartment. In Study-3, rectal sampling provided a uniform site across male and female macaques for *ex vivo* immune assays. This strategy is supported by prior studies showing that intravaginal antiretroviral administration can result in detectable drug and metabolites in rectal tissues and fluids in both macaques and women (46–48). These compartmental differences define the scope of interpretation: the rectal mucosal findings characterize the effects of SAMT-247 and its metabolites on rectal/GI mucosal immunity and provide complementary, although not directly interchangeable, insights into vaginal immune responses.

Met-A is the TPMT-methylated S-derivative of Met-B and lacks antiviral activity (20, 26). The TPMT gene is moderately expressed in hepatocytes (49); therefore, intravaginal delivery may reduce Met-A production. However, the identification of Met-A in vaginal secretions highlights the possible contribution of TPMT by other cells. Low TPMT activity has been detected in human squamous epithelial cells of the vagina (49); however, the relative expression and contribution of TPMT in rhesus macaque vaginal tissue remain to be established. Other thiol methyltransferases, such as TMT1A, may also play a role in generation of Met-A in vaginal tissue. In contrast, tissue-enriched Met-D, interconverting with Met-B and contributing to the active thiol pool (20, 26), may better preserve or amplify ILC3-like and cytolytic NK responses. Additionally, SAMT-247, Met-C, and Met-D also induce the formation of inactivated HIV virions (20), which may further reduce engagement with target cells and indirectly reinforce protective innate responses. Microbicide formulations that maximizing tissue-available Met-D while limiting luminal Met-A could enhance protective mucosal immunity, particularly at the time of viral exposure.

Both immune-cell (7, 24) and proteomic (50) analyses indicated activation-dependent SAMT-247 activity, with broader target engagement following PMA/ionomycin stimulation than under unstimulated conditions. Notably, many of the proteins identified by proteomic profiling were zinc-binding proteins or proteins containing reactive cysteine residues (50). Because SAMT-247 acts through covalent modification of zinc-coordinating cysteine residues, its metabolites may also engage host proteins containing zinc finger motifs or other reactive cysteines, particularly under conditions of immune activation. This context-dependent reactivity may help explain the differential effects of SAMT-247 metabolites on mucosal innate immune populations, with Met-D promoting regulatory and protective signatures, whereas Met-A favors inflammatory responses. These findings suggest that the biological activity of SAMT-247 extends beyond its virucidal properties to include selective modulation of host immune pathways that may influence susceptibility to viral acquisition.

Collectively, these findings demonstrate that SAMT-247 metabolites critically shape mucosal immunity. Met-D most closely recapitulated immune signatures previously associated with reduced SIV acquisition risk, whereas Met-A had the opposite effect, promoting inflammation that may increase susceptibility to viral infection. Taken together, these results highlight the importance of metabolite-specific immune effects in shaping SAMT-247 activity and suggest that future strategies favoring tissue-available Met-D while limiting Met-A could optimize protective mucosal immunity and might improve vaccine efficacy.

## Materials and methods

This research adheres to all relevant ethical guidelines. The study received approval from the National Cancer Institute (NCI) Animal Care and Use Committee (ACUC).

### Animal care, ethics, sample collection, and monitoring

All macaques were housed at the National Institutes of Health (NIH) in accordance with *The Guide for the Care and Use of Laboratory Animals* and the Public Health Service Policy on Humane Care and Use of Laboratory Animals. The NIH facility maintains an active OLAW Assurance (A4149-01) and is accredited by AAALAC International. All study procedures were reviewed and approved by the National Cancer Institute Animal Care and Use Committee (NCI-ACUC).

Animals were observed at least once daily for clinical signs of illness, and veterinary care was provided as needed. Measures to minimize pain and distress were implemented throughout the study. Macaques were socially housed according to the approved ACUC protocol and assessed for social compatibility, except during the viral challenge phase, when individual housing was required. All clinical procedures, including biopsies and administration of anesthetics or analgesics, were performed under the supervision of a laboratory animal veterinarian.

Animals received commercial primate biscuits daily, supplemented with rotating edible enrichment items such as fruits, vegetables, nuts, and forage to support normal growth, maintain body weight, and promote enrichment. Psychological well-being was routinely assessed, and additional enrichment, including sanitized toys, destructible materials, and audio/visual stimuli, was provided.

Samples were collected at prespecified time points before, during, and after the immunization or treatment regimen to assess vaccine-induced immune responses. Depending on the study time point, specimens included peripheral blood, rectal and vaginal pinch biopsies, and rectal and vaginal secretions. When feasible, sample collections were performed concurrently to reduce the number of anesthetic events and recovery periods.

Procedures were conducted under injectable anesthesia to ensure the safety of personnel and animals. The anesthetic regimen was selected based on the procedure and the animal’s body weight and generally consisted of ketamine hydrochloride at 3–25 mg/kg intramuscularly, with or without dexmedetomidine at 7.5-30 µg/kg intramuscularly. Body weight was recorded at each procedure.

Animals were monitored by veterinary technical staff during procedures. The attending veterinarian determined whether continuous monitoring was required based on anesthetic depth and duration, as well as procedural invasiveness. When performed, monitoring included oxygen saturation, heart rate, respiratory rate, end-tidal CO_2_, and body temperature. Animals were not euthanized as part of this study.

### SAMT-247 vaginal tissue and secretion study: study 1

Six female Indian rhesus macaques, 4–5 years of age, were obtained from the free-range breeding colony on Morgan Island, South Carolina. Before enrollment, all animals tested negative for simian immunodeficiency virus (SIV), simian retrovirus type D (SRV-D), simian T-cell leukemia virus (STLV), and Macacine herpesvirus 1, also known as B virus (BV). Screening was performed by serology for SIV, SRV-D, STLV, and BV, and by PCR for SRV-D. Each macaque received a single intravaginal dose of 2 mL SAMT-247 gel, formulated as 0.8% SAMT-247 in hydroxyethylcellulose (HEC) (7). Four hours after dosing, vaginal swabs and vaginal biopsies were collected for metabolite analysis.

### Plasma Met-A and mucosal immune response analysis: study 2

Macaques assigned to the vaccine/S-IVR group were immunized at weeks 0 and 4 with plasmid DNA encoding SIVgp160ΔV1 and SIV239gag. Each DNA immunization consisted of 2 mg SIVgp160ΔV1 DNA and 1 mg SIV239gag DNA formulated in a total volume of 1 mL PBS. The DNA vaccine was administered intramuscularly into both thighs, with 0.5 mL delivered per thigh. At week 8, animals received an ALVAC vector encoding SIV gag/pro/env, containing the wild type env sequence, at a dose of 10^8^ pfu in 1 mL PBS. The ALVAC vaccine was administered intramuscularly into the right thigh. At week 12, animals were boosted with the same ALVAC vector together with SIVgp120ΔV1 protein. The protein boost consisted of 400 μg SIVgp120ΔV1 in 500 μL PBS mixed with 500 μL of 2% Alhydrogel, for a final injection volume of 1 mL. During this boost, ALVAC was administered into the right thigh, whereas the Env protein plus alum formulation was administered into the left thigh. At week 13, animals received a SAMT-247-releasing intravaginal ring (IVR), designed to provide continuous vaginal delivery of the compound. At week 15, plasma and rectal mucosal samples were collected for analysis of plasma Met-A responses and rectal mucosal immune responses (24). Vaginal biopsies were not collected at this time point to avoid disrupting the vaginal mucosa before subsequent SIV challenge; rectal biopsies were therefore used for mucosal immune analyses.

### Rectal tissue samples for *ex vivo* studies: study 3

Rectal tissue samples used for *ex vivo* analyses were obtained from five male and six female rhesus macaques, 3–4 years of age. Animals in that study had previously received either the ΔV1DNA/ALVAC-HIV/ΔV1gp120/alum regimen or the ΔV1DNA/ALVAC-HIV/ΔV1gp120/ALFQA regimen. The alum-adjuvanted group included three males and three females, whereas the ALFQA-adjuvanted group included two males and three females. Rectal tissue samples were collected 32 weeks after the final vaccination and used for subsequent *ex vivo* analyses.

### Vaginal tissue bioanalysis sample preparation

Vaginal tissue biopsies were weighed at the time of collection and immediately flash-frozen in liquid nitrogen. Samples were stored and transported at −80 °C until processing. Prior to enzymatic degradation, tissues were thawed on wet ice for 30 min and subsequently incubated with 500 µL of an enzymatic digestion cocktail composed of RPMI-1640 supplemented with 7.5% (v/v) fetal bovine serum, collagenase (300 U mL^-^¹; LS005275, Worthington Biochemicals, Lakewood, NJ), DNase (2 U mL^-^¹; 18047019, Worthington Biochemicals), elastase (12 U mL^-^¹; LS006363, Worthington Biochemicals), hyaluronidase (60 U mL^-^¹; LS005477, Worthington Biochemicals), and FTC as the internal standard (1.0 µg mL^-^¹). Digestions were performed at 37 °C for 2 h with brief vortex agitation (2 × 5 s) every 15 min.

Following the initial digestion, tissues were mechanically disrupted using a disposable polypropylene microcentrifuge tube pestle (USA Scientific, Ocala, FL). A second 500 µL aliquot of the enzymatic cocktail was added, and samples were incubated for an additional 30 min at 37 °C with intermittent vortex agitation (2 × 5 s every 15 min). Digests were centrifuged at 12,000 rpm for 10 min at 25 °C, and 100 µL of each supernatant was transferred to a V-bottom 96-well plate before being loaded into a 2 mL Impact protein precipitation filter plate (CE0-7565, Phenomenex). Samples were mixed with 300 µL acetonitrile and processed according to the manufacturer’s instructions.

Purified eluates were dried overnight in a SpeedVac concentrator using a medium heat setting. The dried residues were reconstituted in 100 µL of 0.1% (v/v) formic acid in high-purity water, mixed, centrifuged as described above, and analyzed immediately by LC-MS/MS.

### Bioanalysis calibration standards and quality controls

Calibration standards (≥6 concentrations) and quality control (QC) samples (≥3 levels) were prepared in naïve vaginal tissue matrix in accordance with FDA bioanalytical method validation guidelines (51). Due to instability in tissue matrix, standards and QCs for Met-B and Met-C were instead prepared in blank extraction matrix. All standards and QCs were processed in parallel with the pharmacokinetic study samples and analyzed concurrently using liquid chromatography -tandem mass spectrometry (LC-MS/MS).

### Quantification of SAMT-247 and its metabolites in biological samples

SAMT-247 and its metabolites were quantified by LC -MS/MS using a 5 µL injection volume on an HPLC system equipped with a G1367A well-plate autosampler and a G1376A binary pump (1100 Series, Agilent Technologies), coupled to an API 3000 triple quadrupole mass spectrometer (AB Sciex, Framingham, MA) fitted with a Turbo Ion Spray electrospray ionization (ESI) source. Chromatographic separation was achieved on an Agilent Zorbax Eclipse XDB-C18 Rapid Resolution column (2.1 × 50 mm, 3.5 µm) maintained at 40 °C. The mobile phases consisted of (A) 0.1% (v/v) formic acid in water and (B) 0.1% (v/v) formic acid in acetonitrile, delivered at a flow rate of 0.8 mL min^-^¹. The gradient program was as follows: a 4.3-min linear ramp from 100:0 to 65:35 (A:B), followed by a 0.7-min return to 100:0 (A:B), and a 1.5-min hold at 100% A, resulting in a total run time of 6.5 min.

Retention times for the analytes, in order of elution, were: FTC (internal standard), 1.52 min; Met-B, 2.20 min; Met-A, 2.37 min; SAMT-247, 2.42 min; Met-D, 2.53 min; Met-C, 3.11 min. Mass transitions were monitored in positive ESI mode with the following parent → product ion transitions (m/z): SAMT-247, 267.0 → 225.1; Met-A, 239.3 → 222.1; Met-B, 225.3 → 137.1; Met-C, 447.2 → 223.1; Met-D, 223.3 → 209.2; and FTC (IS), 249.0 → 130.0.

Quantification employed ≥8-point calibration curves prepared in blank (unmedicated) biological matrices, except for Met-B and Met-C, for which standards were generated in blank extraction matrix due to instability in tissue matrix. Calibration curves showed linearity with R² > 0.98 for Met-A and Met-D. Accuracy and precision were verified using three independently prepared quality control samples analyzed at the beginning and end of each batch, meeting FDA bioanalytical method validation criteria requiring ≤20% deviation (51).

Analytical measuring ranges for SAMT-247 and its principal metabolites in vaginal tissue samples were defined by the lower limit of quantitation (LLOQ) and the upper limit of quantitation (ULOQ). No analytical measuring range was available for SAMT-247. The measuring ranges were 1–200 ng/sample for Met-A and 91-25,000 ng/sample for Met-D. Samples with analyte concentrations above the ULOQ were diluted into the quantifiable range and reanalyzed to ensure accurate measurement. Concentrations were normalized to biopsy tissue weight and reported as ng mg^-^¹. For cross-analyte comparisons, values were converted from ng mg^-^¹ to µM based on an assumed tissue density of 1 g mL^-^¹.

### *Ex vivo* stimulation assay

The frequencies and cytokine expression profiles of rectal mucosal immune cell subsets were evaluated in 11 rhesus macaques seven months after vaccination with the ΔV1DNA/ALVAC-HIV/ΔV1gp120/alum regimen. Rectal tissue samples were enzymatically and mechanically dissociated into single-cell suspensions, and 1.5 × 10^6^ cells were allocated per stimulation condition. Cells were stimulated with either gp120 (2 μg mL^-^¹), gp120 plus SAMT-247 (1 μM), gp120 plus SAMT-247 Metabolite A (1 μM), gp120 plus SAMT-247 Metabolite B (1 μM), gp120 plus SAMT-247 Metabolite C (1 μM), or gp120 plus SAMT-247 Metabolite D (1 μM). Following antigen stimulation, GolgiPlug protein transport inhibitor (containing Brefeldin A; cat. #555029, 1 μL) and GolgiStop (containing Monensin; cat. #554724, 0.7 μL) were added, and cultures were incubated for an additional 18 h.

After the incubation period, cells were stained with Live/Dead blue dye (cat. #L34962, 0.5 μl; Thermo Fisher), followed by surface staining for 30 minutes at room temperature with the following antibodies: BB700 anti-CD73 (AD2; cat. #750061, 5 μl), APC-Cy7 anti-HLA-DR (L243; cat. #335796, 5 μl), Alexa700 anti-CD3 (SP34-2; cat. #557917, 5 μl), Alexa700 anti-CD20 (2H7; cat. #612905, 5 μl), BV480 anti-CD163 (GHI/61; cat. #333628, 5 μl), BV650 anti-NKp44 (p44-8; cat. #744302, 5 μl), BV786 anti-CD45 (D058-1283; cat. #612905, 5 μl), BUV563 anti-CD16 (3G8; cat. #568289, 5 μl), BUV661 anti-CD141 (1A4; cat. #741650, 5 μl), BUV805 anti-CD14 (M5E2; cat. #612902, 5 μl), and, from BD Biosciences (San Jose, California, USA); PE-Cy7 anti-NKG2A (Z199; cat. #B10246, 5 μl) from Beckman Coulter (Brea, California, USA); AF488 anti-CD1a (O10; cat. #NBP2-34697AF488, 5 μl) from Novus Biologicals (Centennial, Colorado, USA); from Miltenyi Biotec (Bergisch Gladbach, North Rhine-Westphalia, Germany); and PE/Dazzle594 anti-CD16 (3G8; cat. #302054, 5 μl), PE-Cy5 anti-CD11c (3.9; cat. #301610, 5 μl), BV570 anti-CD11b (ICRF44; cat. #301325, 5 μl), BV605 anti-CD1c (L161; cat. #331538, 5 μl), PE anti-MERTK (590H11G1E3; cat. #367608, 5 μl) from BioLegend (San Diego, California, USA). Post surface staining, cells were fixed and permeabilized using the FOX3-transcription buffer set (cat. #00-5523-00; eBioscience, San Diego, California, USA) according to the manufacturer’s instructions.

Subsequently, intracellular staining was performed with the following antibodies: BV750 anti-TNFα (MAb11; cat. #566359, 5 μl), BV421 anti-IL-10 (JES3-9D7; cat. #564053, 5 μl), BUV395 anti-CD107 (H4A3; cat. #565113, 5 μl), BV711 anti-IFN-γ (B27; cat. #5645039, 5 μl) from BD Biosciences (San Jose, California, USA), and PE/Dazzle594 anti-IL-17 (BL168; cat. #512336, 5 μl) from BioLegend (San Diego, California, USA). Samples were acquired on a BD FACSymphony A5 cytometer and analyzed using FlowJo software version 10.6.

Immune cell populations were identified as follows:

- NKG2A^+^ NK cells: singlets/live/CD45^+^/CD3^-^/CD20^-^/CD14^-^/NKG2A^+^/NKp44^-^ (7, 28, 29, 31)
- NKp44^+^ ILCs: singlets/live/CD45^+^/CD3^-^/CD20^-^/CD14^-^/NKG2A^-^/NKp44^+^ (7, 28, 29, 31)
- NKG2A^-^NKp44^-^ ILCs: singlets/live/CD45^+^/CD3^-^/CD20^-^/CD14^-^/NKG2A^-^/NKp44^-^ (7, 28, 29, 31)
- Infiltrating monocytes: singlets/live/CD45^+^/CD3^-^/CD20^-^/CD11b^+^/HLA-DR^+^/FSC-A^low^/CD14^+/-^/CD16^+/-^ (27)
- Myeloid dendritic cells (mDCs): singlets/live/CD45^+^/CD3^-^/CD20^-^/CD14^-^/HLA-DR^+^/CD11c^+^ (27)
- DC-10 subset: singlets/live/CD45^+^/CD3^-^/CD20^-^/HLA-DR^+^/CD1c^-^/CD11b^+^/CD11c^+^/CD14^+^/CD16^+^/CD163^+^/CD141^+^/CD1a^-^ (33)

Population frequencies were reported as percentages of CD45^+^ cells. Cytokine expression was quantified within each respective parent population.

### Rectal mucosal phenotyping in vaccinated animals

The frequencies of natural killer (NK) cells, innate lymphoid cells (ILCs), infiltrating monocytes, and dendritic cells in the rectal mucosa of macaques were measured at two weeks post-IVR insertion (week 15). Eleven freshly collected rectal biopsies were digested with collagenase (2 mg/ml; Sigma-Aldrich) in RPMI (Gibco) without FBS for 1 hour at 37 °C. After incubation, the tissue samples were mechanically disaggregated using a 10 ml syringe with a blunt head cannula, washed with R10 medium, and passed through a 70 μm cell strainer. The cells were counted, and 10–15 million cells were recovered from each biopsy for further analysis.

For phenotype analysis of the rectal mucosa cells, two million cells from each sample were stained with Live/Dead blue dye (cat. #L34962, 0.5 μl; Thermo Fisher) followed by surface staining for 30 minutes at room temperature with the following antibodies: BB700 anti-CD14 (M5E2; cat. #745790, 5 μl), APC anti-CCR2 (48607; cat. #558406, 5 μl), APC-Cy7 anti-HLA-DR (L243; cat. #335796, 5 μl), BV480 anti-CD45 (D058-1283; cat. #566145, 5 μl), BV650 anti-NKp44 (p44-8; cat. #744302, 5 μl), BV750 anti-CD163 (GHI/61; cat. #747185, 5 μl), BUV493 anti-CD73 (AD2; cat. #750061, 5 μl), BUV563 anti-CD184 (CXCR4) (12G5; cat. #741400, 5 μl), BUV661 anti-CD141 (1A4; cat. #741650, 5 μl), BUV737 anti-CD206 (19.2; cat. #741860, 5 μl), BUV805 anti-CD3 (SP34-2; cat. #742053, 5 μl), and BUV805 anti-CD20 (2H7; cat. #612905, 5 μl) from BD Biosciences (San Jose, California, USA); PE-Cy7 anti-NKG2A (Z199; cat. #B10246, 5 μl) from Beckman Coulter (Brea, California, USA); AF488 anti-CD1a (O10; cat. #NBP2-34697AF488, 5 μl) from Novus Biologicals (Centennial, Colorado, USA); PE anti-CD33 (AC104.3E3; cat. #130-113-349, 5 μl) from Miltenyi Biotec (Bergisch Gladbach, North Rhine-Westphalia, Germany); PE/Dazzle594 anti-CD16 (3G8; cat. #302054, 5 μl), PE-Cy5 anti-CD11c (3.9; cat. #301610, 5 μl), BV570 anti-CD11b (ICRF44; cat. #301325, 5 μl), BV605 anti-CD1c (L161; cat. #331538, 5 μl) from BioLegend (San Diego, California, USA). Samples were acquired on a BD FACSymphony A5 cytometer and analyzed using FlowJo software version 10.10.1. The frequency of all cell populations was expressed as a percentage of CD45^+^ cells.

### Rectal mucosal cytokine expression upon gp120 peptides stimulation in vaccinated animals

Two million rectal mucosal cells were stimulated for 2 hours at 37 °C with overlapping gp120 peptides encompassing the sequence of SIVmac251 gp120 (2 μg/ml). Following this initial stimulation, GolgiPlug protein transport inhibitor (containing Brefeldin A) (cat. #555029, 1 μl) and GolgiStop protein transport inhibitor (containing Monensin) (cat. #554724, 0.7 μl) were added, and the cells were cultured for an additional 18 hours. After the incubation period, cells were stained with Live/Dead blue dye (cat. #L34962, 0.5 μl; Thermo Fisher), followed by surface staining for 30 minutes at room temperature. Surface staining was conducted as described above for the phenotyping of mucosal cells. Post surface staining, cells were fixed and permeabilized using the FOX3-transcription buffer set (cat. #00-5523-00; eBioscience, San Diego, California, USA) according to the manufacturer’s instructions. Subsequently, intracellular staining was performed with the following antibodies: R718 anti-TNFα (MAb11; cat. #566957, 5 μl), BV711 anti-IL-10 (JES3-9D7; cat. #564050, 5 μl), BV786 anti-CD107 (H4A3; cat. #563869, 5 μl), BUV395 anti-IFN-γ (B27; cat. #563563, 5 μl) from BD Biosciences (San Jose, California, USA), and BV421 anti-IL-17 (BL168; cat. #512312, 5 μl) from BioLegend (San Diego, California, USA). Samples were acquired on a BD FACSymphony A5 cytometer and analyzed using FlowJo software version 10.10.1. Cytokine expression was gated on the parent population within the mucosal cells.

### SAMT-247 plasma bioanalysis sample preparation

Fresh blood (6–12 ml) was collected at room temperature, centrifuged at 2500 RPM, and the plasma was collected into sterile plasma tubes and stored at -80 °C until analysis. A 100 μL sample, standards, and quality controls (QCs) were crushed with 200 μL of acetonitrile (ACN) containing the internal standard (IS; 75 ng/mL Tolbutamide). The final IS amount in the 300 μL solution was 50 ng. 200 μL of the crushed plasma was dispensed into a 96-well plate and dried in the Biotage TurboVap evaporator (Gas Flow: 55 L/min; Gas Temperature: 55 °C; Plate Temperature: 55 °C; Plate Height: 65 mm; Time: 30minutes). After drying, the residue was resuspended in 75 μL of recon (0.1% FA in 70% H_2_O/30% CAN), vortexed, and centrifuged at 2000 RPM (4 °C) for 10 min before analysis.

### SAMT-247 plasma calibration standard and quality control

Calibration standards (n=8 concentrations, **Table 2**) and QC samples (n=4 levels, **Table 3**) were prepared in naive rhesus macaque plasma (obtained at baseline). Standards were prepared in duplicate sets of 8, and 5 QCs at four levels (LLOQ, LQC, MQC, and HQC) were prepared.

**Table 2:** SAMT-247 plasma LCMS standards.

Standard	X µL of stock	X ul plasma solution	Final volume	Final standard concentration (ng/ml)
A	5	245	250	50
B	5	245	250	25
C	5	245	250	10
D	5	245	250	5
E	5	245	250	2.5
F	5	245	250	1
G	5	245	250	0.5
H	5	245	250	0.25

**Table 3:** SAMT-247 plasma LCMS QCs.

QC level	Desired QC conc. (ng/mL)	Stock used	X µL of stock	Plasma (ul)	Final volume (µL)
LLOQ	0.25	G (25 ng/mL)	6.0	594.0	600.0
LQC	0.75	F (50 ng/mL)	9.0	591.0	600.0
MQC	20	B (1250 ng/mL)	9.6	590.4	600.0
HQC	40	A (2500 ng/mL)	9.6	590.4	600

### Quantification of plasma SAMT-247 metabolites

Plasma concentrations of SAMT-247 Met-A in rhesus macaque plasma were quantified using a validated LC-MS/MS method. Samples were analyzed using a 1 µL injection volume on a Waters ACQUITY UPLC I-Class System equipped with an autosampler and coupled to an AB Sciex QTRAP 6500 mass spectrometer (AB Sciex Pte. Ltd., Singapore). Chromatographic separation was achieved on an Acuity UPLC BEH Shield RP18 column (2.1x50 mm, 1.7μm). The autosampler was maintained at 4 °C. Mobile Phase A consisted of 0.1% v/v formic acid in water and mobile phase B consisted of 0.1% v/v formic acid in acetonitrile, at a flow rate of 0.6 mLmin^-1^ using the following gradient: 0.0-0.2 min, 95:5 A:B to 80:20 A:B; 0.1-1.0 min, and hold at 80:20 A:B; 1.0-1.1 min, 80:20 A:B to 65:35 A:B; 1.1-1.2 min, 65:35 A:B to 5:95 A:B; 1.2-2.2 min, hold at 5:95 A:B, 2.2-2.3 min, return to 95:5 A:B; and 2.3-2.5 min, re-equilibrate at 95:5 A:B for a total run time of 2.5 min. SAMT-247 metabolite A and internal standard (IS) retention times were 0.66 and 1.5 minutes, respectively. Detection was performed in ESI+ ionization mode using multiple reaction monitoring. The monitored transitions were m/z 267.0 → 225.1 for SAMT-247, m/z 239.3 → 222.1 for SAMT-247 Met-A, and m/z 271.0 → 155.0 for the internal standard. Data acquisition and quantitative analysis were performed using SCIEX OS version 1.6.1. For acceptance of a calibration curve, at least 75% of non-zero calibration standards, with a minimum of six concentration levels, were required to be within ±15% of nominal concentrations, except at the lower limit of quantitation, where ±20% was permitted (**Table 4**). Within-run and between-run accuracy and precision were established using at least three independent validation runs with at least five replicates per QC level, including lower limit of quantitation, low, mid, and high QCs. Accuracy at each standard and QC level was required to be within ±15% of nominal concentration, except at the lower limit of quantitation where ±20% was acceptable (**Table 4**). Precision was required to be ≤15% CV at each QC level, except at the lower limit of quantitation where ≤20% CV was acceptable (**Table 4**). During study sample analysis, each analytical run included calibration standards and QC samples at low, mid, and high concentrations. Run acceptance required that at least two-thirds of all QC samples and at least 50% of QC samples at each concentration level be within ±15% of nominal values. Carryover, dilution integrity, and stability were evaluated during validation and were acceptable for study sample analysis.

**Table 4:** Analytical measuring range (ng/ml).

Analyte	Plasma range (LLOQ - HQC)	% accuracy (calibrators)	% CV (precision)	Coefficient of determination (R^2^)
Met-A	0.25–50 ng/ml	Standards/QC +or- 15% LLOQ : +or- 20%	Standards/QC: ≤ 15 LLOQ : ≤ 20	0.99

Range is defined by the lower limit of quantitation (LLOQ) and the upper limit of quantitation (HQC).

### Statistical analysis

All statistical analyses were performed using non-parametric methods, as no assumptions were made regarding normality or homogeneity of variance. Comparisons of continuous variables between two paired groups were conducted using the two-tailed Wilcoxon signed-rank test. Because the study was exploratory, P values are reported as nominal and were not adjusted for multiple comparisons. No animals or data points were excluded from the analyses.

## Data Availability

The original contributions presented in the study are included in the article/**Supplementary Material**. The code to produce all figures can be found at https://github.com/NCI-VB/rahman_woode_samtMetabolites. Further inquiries can be directed to the corresponding authors.
