# Supplementary material for: Friend or foe: divergent immunomodulatory effects of metabolites derived from the virucidal zinc finger inhibitor SAMT-247
Source: Front Immunol. 2026 Aug 20;17:1879043. doi: 10.3389/fimmu.2026.1879043 (PMC13538096; doi:10.3389/fimmu.2026.1879043)
Supplement: Supplementary file 1 [file SupplementaryFile1.docx]

**Supplementary Figure 1 | Effects of SAMT-247 and Met-B and Met-C derivatives on mucosal NK/ILC responses. A.** Heatmaps depict stimulation-induced (gp120 response was subtracted from SAMT-247+gp120/ Met-B+gp120/ Met-C+gp120) changes in NK/ILC responses. Note same heat scale is used across all analytes to enable comparison of both magnitude and direction. Comparison between SAMT-247+gp120/ Met-B+gp120/ Met-C+gp120 stimulation vs gp120 alone for NK/ILC responses were performed. Alluvial flow connects each population across the different stimulation. **B-G.** Immunomodulatory effects of SAMT-247, Met-B, and Met-C on NK/ILC subsets in rectal mucosa from 11 vaccinated macaques, y-axes indicate delta in percentage for each analyte (gp120 response was subtracted from SAMT-247+gp120 or Met-B+gp120 or Met-C+gp120): **B.** CD73⁺NKp44⁺ ILCs; **C.** CD73⁺NKG2A⁺ NK cells; **D.** CD73⁺NKG2A⁻NKp44⁻ ILCs; **E.** CD107⁺NKG2A⁺ NK cells; **F.** NKp44⁺ ILCs; and **G.** NKG2A⁻NKp44⁻ ILCs. Data were analyzed using two-sided paired non-parametric Wilcoxon signed Rank test. Box-and-whisker plots show minimum, Q1, median, Q3, and maximum values.

**Supplementary Figure 2 | Effects of SAMT-247 and its derivatives on mucosal dendritic cell responses. A.** Heatmaps depict stimulation-induced (gp120 response was subtracted from SAMT-247+gp120/ Met-B+gp120/ Met-C+gp120) changes in mDC and DC10 responses. Note same heat scale is used across all analytes to enable comparison of both magnitude and direction. Comparison between SAMT-247+gp120/ Met-B+gp120/ Met-C+gp120 stimulation vs gp120 alone for mDC and DC10 responses were performed. Alluvial flow connects each population across the different stimulation. **B-F.** Immunomodulatory effects of SAMT-247 and its metabolites on mucosal DC subsets from 11 vaccinated macaques, y-axes indicate delta in percentage for each analyte (gp120 response was subtracted from SAMT-247+gp120 or Met-B+gp120 or Met-C+gp120): **B.** IL-10⁺ DC10; **C.** CD73⁺ DC10; **D.** IL-10⁺ mDC; **E.** DC10; and **F.** TNF-α⁺ mDC. Data were analyzed using two-sided paired non-parametric Wilcoxon signed Rank test. Box-and-whisker plots show minimum, Q1, median, Q3, and maximum values.

**Supplementary Figure 3 | Effects of SAMT-247 and its metabolites on mucosal infiltrating monocyte responses. A.** Heatmaps depict stimulation-induced (gp120 response was subtracted from SAMT-247+gp120/ Met-B+gp120/ Met-C+gp120) changes in infiltrating monocyte responses. Note same heat scale is used across all analytes to enable comparison of both magnitude and direction. Comparison between SAMT-247+gp120/ Met-B+gp120/ Met-C+gp120 stimulation vs gp120 alone for infiltrating monocyte responses were performed. Alluvial flow connects each population across the different stimulation. **B-D.** Immunomodulatory effects of SAMT-247 and its derivatives on monocyte subsets in rectal mucosa from 11 vaccinated macaques, y-axes indicate delta in percentage for each analyte (gp120 response was subtracted from SAMT-247+gp120 or Met-B+gp120 or Met-C+gp120) : **B.** CD73⁺CD14⁺CD16⁻ monocytes; **C.** IL-10⁺CD14⁺CD16⁻ monocytes; and **D.** IFN-γ⁺CD14⁻ CD16⁺. Data were analyzed using two-sided paired non-parametric Wilcoxon signed Rank test. Box-and-whisker plots show minimum, Q1, median, Q3, and maximum values.

**Supplementary Figure 4 | Correlation analysis of plasma Met-A with mucosal immune responses.** Correlation of concentration of Met-A in plasma with different mucosal immune responses post *in vivo* SAMT-247 treatment by IVR. Data were analyzed using two-sided Spearman correlation.

**Supplementary Figure 5 | Summary of immune responses induced by SAMT-247 and its metabolites.** This figure summarizes the innate immune responses induced by SAMT-247 and its metabolites in modulating mucosal immune responses.
